# CCDC65, a Gene Knockout that leads to Early Death of Mice, acts as a potentially Novel Tumor Suppressor in Lung Adenocarcinoma

**DOI:** 10.7150/ijbs.69332

**Published:** 2022-06-27

**Authors:** Ziyan Zhang, Ping Xu, Zhe Hu, Zhaojian Fu, Tongyuan Deng, Xiaojie Deng, Lanzhu Peng, Yingying Xie, Lingzhi Long, Dayong Zheng, Peng Shen, Mengmin Zhang, Bin Gong, Zhibo Zhu, Junhao Lin, Rui Chen, Zhen Liu, Huilin Yang, Rong Li, Weiyi Fang

**Affiliations:** 1 Cancer Center, Integrated Hospital of Traditional Chinese Medicine, Southern Medical University, 510315 Guangzhou, China.; 2 Cancer Research Institute, Southern Medical University, Guangzhou, Guangdong, 510515, China.; 3 Respiratory Department, Peking University Shenzhen Hospital, Shenzhen, 518034, China.; 4 Department of Oncology, Dali Bai Autonomous Prefecture People's Hospital, Dali, Yunnan, 671000, China.; 5 Department of Pulmonary and Critical Care Medicine, The Third Xiangya Hospital of Central South University, Changsha, 410000, China.; 6 Department of Oncology, Nanfang Hospital, Southern Medical University, 510515 Guangzhou, China.; 7 Department of Hepatobiliary Surgery, The First Affiliated Hospital of Xi'an Jiaotong University, Xi'an, 710061, China.; 8 School of Pharmacy, Guangdong Medical University, Dongguan, Guangdong 523808, China.; 9 Affiliated Cancer Hospital & Institute of Guangzhou Medical University, Guangzhou Municipal and Guangdong Provincial Key Laboratory of Protein Modification and Degradation, State Key Laboratory of Respiratory Disease, School of Basic Medical Sciences, Guangzhou Medical University Guangzhou 510095, Guangdong, China.

**Keywords:** CCDC65, c-Myc, ENO1, Knockdown mice, Ubiquitin degradation, Cell cycle

## Abstract

CCDC65 is a member of the coiled-coil domain-containing protein family and was only reported in gastric cancer by our group. We first observed that it is downregulated in lung adenocarcinoma based on the TCGA database. Reduced CCDC65 protein was shown as an unfavorable factor promoting the clinical progression in lung adenocarcinoma. Subsequently, CCDC65^-/-^ mice were found possibly dead of hydrocephalus. Compared with the CCDC65^+/+^ mice, the downregulation of CCDC65 in CCDC65^+/-^ mice significantly increased the formation ability of lung cancer induced by urethane. In the subsequent investigation, we observed that CCDC65 functions as a tumor suppressor repressing cell proliferation *in vitro* and *in vivo*. Molecular mechanism showed that CCDC65 recruited E3 ubiquitin ligase FBXW7 to induce the ubiquitination degradation of c-Myc, an oncogenic transcription factor in tumors, and reduced c-Myc binding to ENO1 promoter, which suppressed the transcription of ENO1. In addition, CCDC65 also recruited FBXW7 to degrade ENO1 protein by ubiquitinated modulation. The downregulated ENO1 further reduced the phosphorylation activation of AKT1, which thus inactivated the cell cycle signal. Our data demonstrated that CCDC65 is a potential tumor suppressor by recruiting FBWX7 to suppress c-Myc/ENO1-induced cell cycle signal in lung adenocarcinoma.

## Introduction

Lung cancer has a high incidence and the highest mortality among tumors. In recent years, adenocarcinoma has become the most common type among all types of lung cancer 1. Currently, the treatment of lung adenocarcinoma (LUAD) has gradually become “individualized”. Through the detection of specific gene mutations, patients can be treated with precision therapy using personalized treatments, such as the use of EGFR-TKI drugs in EGFR mutation NSCLC. Targeting the EGFR 2, 3 has been proven to have a longer median duration of progression-free survival compared to traditional chemotherapy. Thus, exploring novel important biomarkers of LUAD could significantly improve the prevention, diagnosis, and treatment of LUAD.

The coiled-coil domain (CCD)-containing (CCDC) family plays a key role in multiple important biological functions such as regulating T cell activity 4, motile cilia function 5, erythroid iron levels, heme trafficking 6, and DNA sensors 7. Meanwhile, CCDC also modulates the development of malignant tumor proliferation and metastasis 8-11.

In our previous research, we cloned and revised the coding sequence of NESG1 (also known as CFAP45 and CCDC19) and subsequently confirmed that it acts as a potential tumor suppressor in NPC and non-small cell lung cancer 12-14. Yeast Two-Hybrid experiments showed that CCDC65 was one of the candidates interacting proteins. Although the further experiment confirmed that CCDC65 did not interact with NESG1. Interestingly, CCDC65 was found significantly downregulated in nasopharyngeal carcinoma and lung cancer.

CCDC65, known as CFAP250, DRC2, FAP250, and NYD-SP28, is located on the long arm of chromosome 12. It is firstly identified in the sperm tail 15. Subsequently, the specific functions of CCDC65 revealed that it was an essential component of the nexin-dynein regulatory complex participating in the formation of motile cilia. Mutations in CCDC65 caused primary ciliary dyskinesia (PCD), a disease characterized by impaired ciliary function leading to chronic sinopulmonary disease 16, 17. In tumor areas, only our research group recently reported that CCDC65 played the role of a tumor suppressor in gastric cancer 18. However, the role of CCDC65 in LUAD remains unclear.

Here, we detected the expression of CCDC65 in LUAD, evaluated the tumor suppressor role of CCDC65 in human LUAD cells, CRISPR/Cas9 mouse model and nude mice.

## Materials and methods

### Cell culture and reagents

Human lung adenocarcinoma cancer cell lines used were: A549, H1299, H1975, SPC-A1 and H460. Human normal bronchial epithelial cell line used was 16HBE. Lung adenocarcinoma cancer cell lines were cultured in RPMI 1640 Medium (Vivacell, Shanghai, China) containing 10% fetal bovine serum (FBS) (Biowest, Nuaillé, France), HEK293T and 16HBE were cultured in DMEM (Vivacell, Shanghai, China) containing 10% FBS (Biowest, Nuaillé, France) at 37 °C in a 5% CO_2_ atmosphere. Culture dishes were purchased from Jet Biofil (Guangzhou, China).

### Western blotting and antibodies

Western blotting was performed according to standard methods. Antibodies of CCDC65 (24376-1-AP), ENO1 (11204-1-AP), c-Myc (10828-1-AP), CCND1 (60186-1-Ig), p-AKT (Ser473) (66444-1-Ig), DYKDDDDK Tag (80010-1-RR), mTOR(66888-1-Ig), p-mTOR(Ser2448) (677778-1-Ig), GSK3β(22104-1-AP), p-GSK3β(Ser9) (67558-1-Ig), were purchased from Proteintech (Rosemont, IL, USA). Anti-P-Akt1 (Ser473) (#9018), Mouse IgG (#3420), normal Rabbit IgG (#2729), Anti-p27(#3686), Anti-p21(#2947) were purchased from Cell Signaling Technology (Danvers, MA, USA). Anti-AKT1 (48884) was purchased from Signalway Antibody (College Park, MD, USA). Anti-flag (F1804) was purchased from Sigma (St. Louis, MO, USA); Anti-β-actin (AP0060) and anti-GAPDH (AP0063) were purchased from Bioworld (Bloomington, MN, USA). Antibodies were diluted with SuperKine™ Enhanced Antibody Dilution Buffer (Abbkine, Wuhan, China). Western blotting was performed by Mini-PROTEAN Tetra- and Mini Trans-Blot (Bio-Rad, Hercules, CA, USA). The images were captured with Chemiluminescence Imaging System (Minichemi, Beijing, China).

### Reverse transcription and quantitative real-time polymerase chain reaction (RT-qPCR)

Total RNA from cells and animal tissues were extracted using the Total RNA Isolation Kit (Foregene, Chengdu, China). Using Reverse Transcription Reagent Kit (Vazyme, Nanjing, China), mRNA was transcribed into cDNA, which acted as a template to amplify target genes in subsequent qPCR tests. Quantitative real-time PCR was detected by CFX96 Real-Time System (Bio-Rad). Primers are shown in supplementary materials (Supplementary Table S1).

### Tissue samples and immunohistochemical staining

Seven human lung adenocarcinoma and corresponding para-carcinoma tissue specimens as well as twenty cases of un-paired para-carcinoma tissues were obtained from TCM-Integrated Hospital of Southern Medical University. The use of clinical materials was approved by the Ethics Committees of TCM-Integrated Hospital of Southern Medical University. Tissue microarray (HLugA180Su08) was purchased from Outdo Biotech (Shanghai, China). Streptavidin/Peroxidase kit (ZSGB BIO, Beijing, China) and DAB kit (ZSGB BIO) were used for immunohistochemical staining. Immunohistochemical staining was performed according to standard methods. Scoring rule: multiply the score of staining intensity by the score of the proportion of positive cells (0-12 points). Proportion score: Positive cells less than 25% (0 points), 25-50% (1 points), 50-75% (2 points), 75% above (3 points). Strength score: - (0 points), + (1 points), ++ (2 points), +++ (3 points). Total score: 0-6 scores (low expression), 7-12 scores (high expression). Staining scores were performed with the assistance of two senior doctors in the Department of Pathology.

### Genotyping

Mice were genotyped by DNA sequence and standard PCR. Primers were shown in supplementary materials (Supplementary Table S1). Agarose gel was prepared with 2% agarose and TAE (Leagene, Beijing, China). The PCR product was mixed with DNA loading buffer and Gelred (Biosharp, Anhui, China). After electrophoresis, the images were captured with GelDoc XR^+^ (Bio-Rad).

### Lung adenocarcinoma model mouse and HE staining

C57BL/6J CCDC65^+/-^ mice were purchased from Nanjing BioMedical Research Institute of Nanjing University (Nanjing, China). CCDC65 engineered mice were constructed by CRISPR/Cas9 technology, gRNA was designed and transcribed *in vitro*. The fertilized eggs of mice were simultaneously injected with Cas9 and all 4 gRNA within half a day. After embryo transfer, F0 generation mice were born about 19 days later. C57BL/6J wild-type mice were purchased from Animal Research Center of Southern Medical University (Guangzhou, China). Mice were housed and bred in a specific pathogen-free (SPF) environment. Mice were divided into 2 groups: CCDC65^+/+^ and CCDC65^+/-^, 15 mice per group (6-8 weeks old, 8 males and 7 females). The lung adenocarcinoma mouse model was achieved by intraperitoneal injection of urethane (1 g/kg in 0.9% NaCl solution, Sigma) once a week for 20 weeks 19. Mice were sacrificed and lungs were harvested 20 weeks after the end of urethane treatment. The tissues were dehydrated, paraffin-embedded, and prepared into 3-μm paraffin sections. Slides were dewaxed using xylene and gradient alcohol solutions and then treated with hematoxylin for 5 min and eosin for 1 min. After dehydration, the slices were sealed.

### Transfection

When cells reached 40% confluence, transfection was performed using Lipofectamine 2000 Transfection Reagent (Invitrogen, Carlsbad, CA, USA) according to the operating instructions. The RNA of cells were harvested 24-48 hours after transfection and protein of cells were harvested 48-72 hours after transfection.

### Lentivirus infection

The full-length sequences of CCDC65 and FLAG-Tag were inserted into the lentivirus (Genechem, Shanghai, China) which contains green fluorescent protein (GFP). To build stably over-expressing CCDC65 cell lines, A549, H1975, and SPC-A1 were infected with GFP empty vector or full-length CCDC65 GFP vector.

### Cell Counting Kit 8 assay

Cells were seeded into 94-well plates at 3000 cells/well, and each condition was performed in quadruplicate. Cell Counting Kit (CCK8) (Vazyme) was added every 24 hours for 4 days and after being treated with CCK8 for 2 hours, the optical density (OD) value of each well was measured at 450 nm.

### Colony formation assay

Cells were seeded into 6-well culture plates at 200 cells/well. Cells were incubated for two weeks at 37 °C in a 5% CO_2_ atmosphere and the medium was changed every 4 days. After incubation, colonies were washed twice with PBS and fixed with methanol, and finally stained with Giemsa.

### 5-Ethynyl-2'-deoxyuridine staining

Cells were seeded into 96-well plates at 5000 cells/well and experiments were performed in triplicate for each condition in each group. At 12 hours after seeding, the culture medium was changed to FBS-free or low FBS medium for 24 hours to synchronize the cell cycle. 5-Ethynyl-2'-deoxyuridine (EdU) staining was performed using the Cell-Light EdU Apollo567 *in vitro* Kit (Ribobio, Guangdong, China) and 4',6-Diamidino-2-phenylindole dihydrochloride (DAPI) (Beyotime Biotechnology, Shanghai, China) according to manufacturer's instructions.

### Cell-cycle analysis

A total of 10^7^ cells were harvested 48 hours after transfection and washed twice with PBS. The cells were fixed with 70% ethanol. Then, cells were treated with 10 mg/mL propidium iodide (PI) and 0.5 mg/mL RNase A for 15 min at 37 °C. After washing three times with PBS, A FACS caliber flow cytometer (BD Biosciences) was used to measure the DNA content of treated cells.

### Tumor xenograft experiments

Four-week-old nude mice were purchased from SPF (Beijing) Biotechnology Co., Ltd. (Beijing, China). The mice were housed in the SPF environment. Cells were infected with lentiviral vectors encoding CCDC65 or control vectors. A total of 5×10^6^ cells were placed into 100 μL PBS. Controls cells were subcutaneously injected into the left back and CCDC65 infected cells were subcutaneously injected into the right back. After 28 days, the mice were sacrificed and tumor nodules were weighed, dehydrated, and paraffin-embedded.

### Co-immunoprecipitation assay

Cells were treated with immunoprecipitation (IP) lysis buffer (Thermo Scientific, Waltham, Mass, USA). Cell lysates were exposed to specific antibodies or Normal IgG (final concentration of 20 μg/mL) at 4 °C overnight and stirred using a rotator. The protein-antibody compound followed incubation with protein A/G magnetic beads (Bimake, Shanghai, China) for 30 min at room temperature. The precipitate was washed with washing buffer (50 mM Tris, 150 mM NaCl, 0.2% TritonX-100, 0.2% Tween, pH 7.5) five times. The bound proteins were resolved in SDS loading buffer and boiled for 10 minutes and subjected to immunoblotting.

### Immunofluorescence staining

Cells were harvested 48 hours after transfection, seeded into laser confocal petri dishes for 12 hours, and then washed twice with PBS. The cells were then fixed with paraformaldehyde and 2 mg/mL glycine for 15 min and permeabilized with 0.2% Triton X-100 for 5 min. After blocking with 5% BSA for 30 min and incubating with specific primary antibodies at 4°C overnight, the cells were washed three times with PBST and stained with Alexa Fluor secondary antibodies (Earthox, Millbrae, CA, USA) and DAPI (Beyotime, Biotechnology).

### Cycloheximide chase assay and ubiquitination assay

The cycloheximide (CHX) chase assay and ubiquitination assay were performed as described previously 20, 21. Cells were treated with CHX (50 μg/mL) (Selleck, Shanghai, China) for Gradient time. Proteins were extracted using RIPA lysis buffer and mixed with SDS loading buffer and boiled for 10 minutes, and then were subjected to immunoblotting. For the ubiquitination assay, cells were treated with MG132 (20 μM) (Selleck) for 4 hours before the proteins were harvested. The proteins were extracted using IP lysis buffer and immunoprecipitation was performed with anti-c-Myc or anti-ENO1. Harvested proteins were resolved in SDS loading buffer and boiled for 10 minutes, then subjected to immunoblotting.

### High-throughput sequencing and bioinformatics analysis

High-throughput mRNA sequencing and subsequent bioinformatics analysis between CCDC65^+/+^ and CCDC65^+/-^ mice lung tissues were performed by the Oebiotech Biomedical Technology Co., Ltd (Shanghai, China). The sequencing and analysis in A549, SPC-A1 and 5-8F were performed by Genergy Inc (Shanghai, China). Detailed transcriptome mice sequencing information was available in the SRA database (PRJNA695129).

### Chromatin immunoprecipitation assay

The experiments were performed with Pierce Magnetic ChIP Kit and according to the manufacturer's manual (Thermo Scientific, Waltham, Mass, USA). Briefly, cells were cross-linked by the final concentration of 1% formaldehyde. Cross-linked cells were treated with membrane extraction buffer and MNase to remove membrane proteins and expose the nucleic acid. Four micrograms of c-Myc antibody and 2 μg normal rabbit IgG antibody were added to the supernatant containing the digested chromatin, as the experimental group and control group respectively, and IP reactions were incubated overnight at 4 °C with mixing. After incubation, ChIP grade protein A/G magnetic beads were added to each IP reaction and further incubated for 2 hours at 4 °C with mixing. Cleaned the beads and eluted the nucleic acid-proteins complex among the beads. Proteinase K was used to remove the proteins in the nucleic acid-proteins complex. DNA fragments were purified after being washed in the DNA clean-up column. JASPAR database was used to predict promoter binding sequences 22.

### Statistical analysis

Statistical analyses were performed with the IBM SPSS Statistics 25 (IBM, Armonk, NYC, USA). Comparisons between two groups were performed using two independent samples t-test. Comparisons between multiple groups were performed using one-way ANOVA. Survival plots were performed using the log-rank test. Cox regression was used to screen out single and multiple risk/protective factors. Spearman correlation coefficient was used for grade data analysis. All statistical tests were two-sided and the asterisk indicated statistical significance. *P<0.05, **P<0.01, ***P<0.001.

## Results

### CCDC65 was downregulated in human LUAD and correlated with the clinical characteristics of LUAD patients

**Figure 1:**
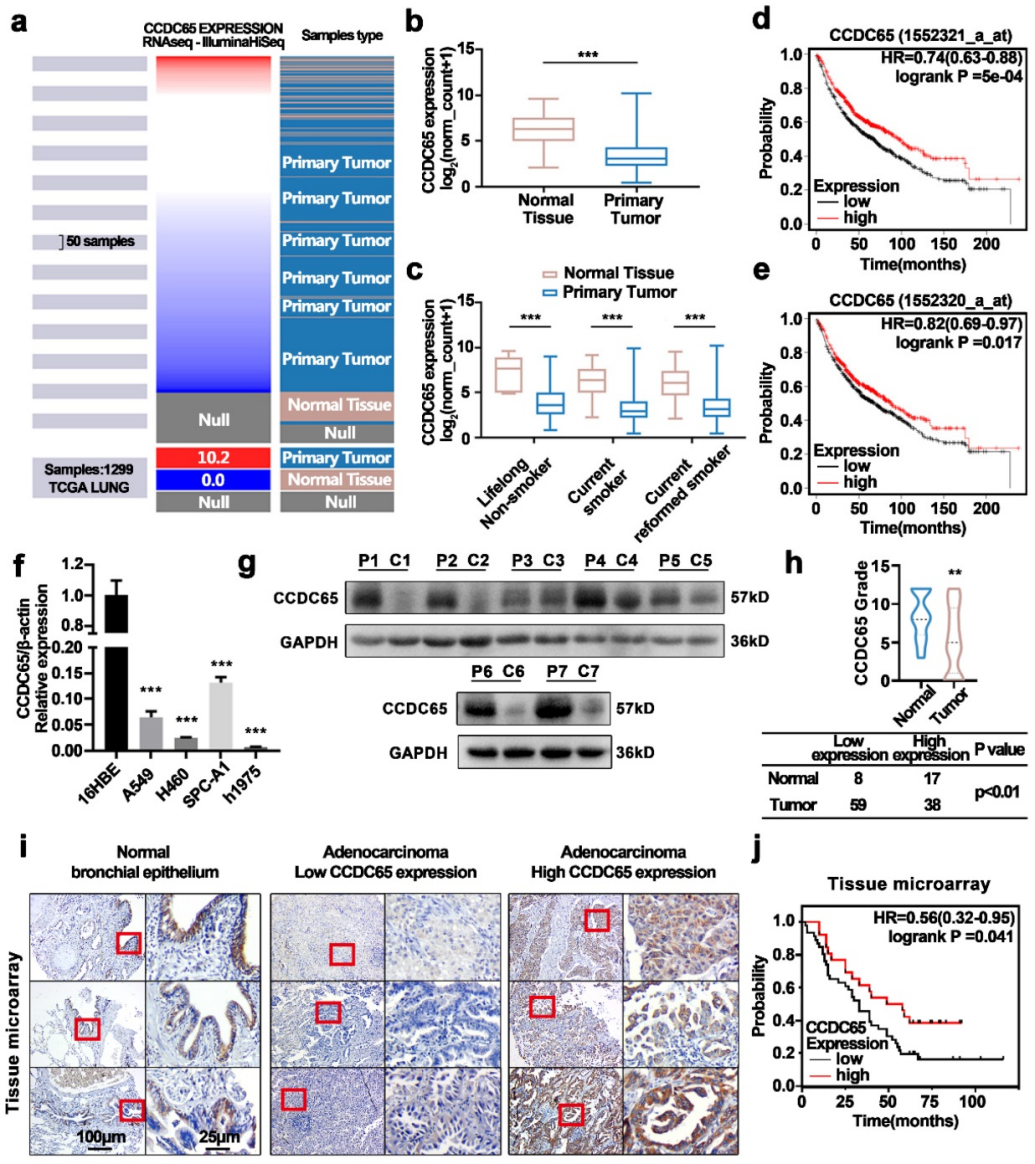
** Higher expression of CCDC65 inhibited lung adenocarcinoma and was associated with a better prognosis. (a)** The heatmap of CCDC65 mRNA expression was derived based on samples from the TCGA lung cancer database (n =1299). **(b)** Comparison of the expression of CCDC65 mRNA in primary tumors and normal tissues. **(c)** Comparison of the expression of CCDC65 mRNA in primary tumors and solid normal tissues stratified according to smoking history. The data were analyzed using UCSC Xena (http://xena.ucsc.edu/). Kaplan-Meier survival curves relative to CCDC65 expression were generated from lung cancer (Kaplan-Meier plotter, http://kmplot.com/analysis). The Affymetrix IDs validated were **(d)** 1552321_a_at CCDC65 and **(e)** 1552320_a_at CCDC65. **(f)** qRT-PCR analysis of CCDC65 expression in lung adenocarcinoma cell lines and immortalized human bronchial epithelium cell line. **(g)** Expression of CCDC65 protein in 7 paired lung adenocarcinoma and adjacent nontumor tissues were detected by western blotting. P: para-carcinoma tissues C: cancer tissues. **(h)** The CCDC65 expression in lung cancer and para-carcinoma tissues (20 cases of para-carcinoma tissues from clinical samples and 5 cases of para-carcinoma tissues from tissue microarray, 97 cases of lung cancer from tissue microarray). **(i)** Tissues microarray immunohistochemical analysis of CCDC65 protein expression. **(j)** The survival curve was determined by Kaplan-Meier analysis based on the tissue microarray immunohistochemical score.

Analysis of The Cancer Genome Atlas (TCGA) lung cancer database showed that CCDC65 was downregulated in primary lung tumors (Fig. 1a, b). Due to CCDC65 being a component of cilia, which is always affected by smoking, we divided datasets from TCGA into three groups based on the smoking status (Fig. 1c). The results showed that CCDC65 was expressed at lower levels in primary lung tumor samples compared to normal lung tissues, regardless of smoking history. We then constructed survival plots using the KM plotter 23 (Fig. 1d, e). The results showed that the survival probability of patients with high CCDC65 expression was higher than patients with low CCDC65 expression. Subsequently, we detected the mRNA expression of CCDC65 in a normal bronchial epithelial cell line and four LUAD cell lines (Fig. 1f). Furthermore, the protein expression of CCDC65 in seven human LUAD and corresponding para-carcinoma tissue were also compared (Fig. 1g). Immunohistochemistry staining was performed in tissue microarray and clinical tissue samples (Fig. 1h, 1i). Statistical analysis was used in immunohistochemical staining scores to compare the expression of CCDC65 among different sample types. The results showed that CCDC65 was significantly downregulated in LUAD both in cell lines and human tissues.

Based on the detailed clinical data associated with the tissue microarray samples IHC scores, One Way ANOVA analysis revealed that the expression of CCDC65 was associated with N stage (P=0.041) in LUAD patients (Table 1). The univariate Cox regression analysis showed that lymph node metastasis (P=0.002 HR=3.22 95%CI=1.517-6.835), T stage (P=0.028 HR=1.509 95%CI=1.044-2.180), N stage (P<0.001 HR=1.437 95%CI=1.310-2.303), and the expression of CCDC65 (P=0.047 HR=0.551 95%CI=0.306-0.993) were associated with the patients' survival. Multivariate Cox regression analysis showed that the expression of CCDC65 was an independently protective prognostic factor (P=0.049 HR=0.513 95%CI=0.264-0.998) (Table 2). Finally, we drew survival plots based on the information from the tissue microarray (Fig. 1j). The results were in line with the database and indicated that the survival probability of patients with high CCDC65 expression was better than patients with low CCDC65 expression.

### The CCDC65^-/-^ mice experienced early mortality probably due to hydrocephalus

CCDC65 engineered mice were generated by CRISPR/Cas9 technology and the strategies were presented in supplementary materials (Supplementary Fig. S1a). CCDC65^+/-^ mice were allowed to copulate (female: male=2:1). DNA was extracted from the mouse tail at the age of 1 week for genotyping (Supplementary Fig. S1b). We detected the mRNA and protein expression of CCDC65 in the lung tissue of different groups of mice, and the results identified the knockout of CCDC65 (Supplementary Fig. S1c-e).

Mice were then divided into three groups: CCDC65^+/+^, CCDC65^+/-^, and CCDC65^-/-^. And the sex ratio and genotype ratio (Supplementary Fig. S2a) were determined six weeks after the mice were born. Results showed that the birth rate of CCDC65^-/-^ mice was lower than the expected rate from Mendelian law. The CCDC65^-/-^ mice died at three weeks of age (Supplementary Fig. S2b) and developed slowly compared with the CCDC65^+/+^ mice or CCDC65^+/-^ mice (Supplementary Fig. S2c, S2d). The mice were dissected to explore the reason. Except for the possible cardiac transposition heart (red arrow), the lung, liver, kidney, stomach, and intestines of CCDC65^-/-^ mice showed no significant differences compared with CCDC65^+/+^ mice (Supplementary Fig. S2e). Remarkably, the morphological structure of brain tissue in the CCDC65^-/-^ group was significantly different from that in the CCDC65^+/+^ and CCDC65^+/-^ groups. The craniocerebral diameter ratio of mice in the CCDC65^-/-^ group was significantly lower than that in CCDC65^+/+^ and CCDC65^+/-^ mice (Supplementary Table S2), and the brain tissue of mice in the CCDC65^-/-^ group was sunken, while the brain tissue of mice in CCDC65^+/+^ group and CCDC65^+/-^ the group was full. Similar changes were observed from mice raised in different cages (Supplementary Fig. S2f, g, h). Paraffin-embedded brain tissue and HE staining revealed that the lateral ventricle of CCDC65^-/-^ mice were significantly larger than that in the CCDC65^+/+^ mice or CCDC65^+/-^ mice (Supplementary Fig. S2i). Based on the known functions of CCDC65 in cilia^16^, ^17^, we supposed that CCDC65^-/-^ died of hydrocephalus. However, this was not the main point we focused on, therefore, in-depth research was not performed.

The mice in the CCDC65^-/-^ group did not live more than three weeks. However, the mRNA levels of CCDC65 in the CCDC65^+/-^ group decreased significantly compared with the CCDC65^+/+^ group, even at different ages (Supplementary Fig. S1c). And the protein expression also decreased (Supplementary Fig. S1d, S1e). Consequently, CCDC65^+/+^ and CCDC65^+/-^ mice were used to reveal the function of CCDC65 in tumorigenesis.

### The downregulation of CCDC65 promoted lung tumorigenesis *in vivo*

To investigate the role of CCDC65 in LUAD, we used a mouse model of urethane-induced LUAD to confirm our hypothesis 19, 24, 25.

**Figure 2:**
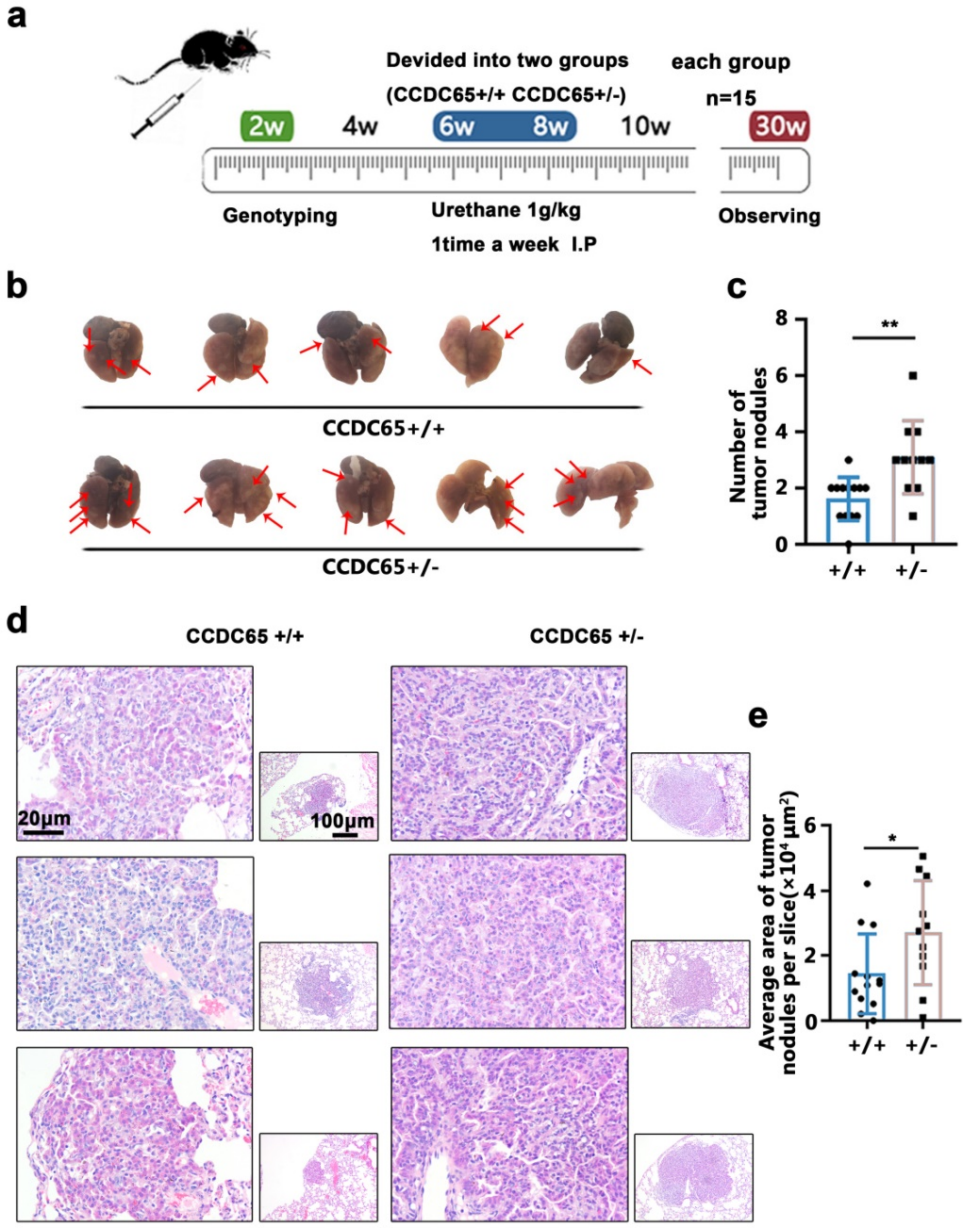
** CCDC65 inhibited urethane-induced lung carcinogenesis. (a)** The mice were genotyped at the age of two weeks and then CCDC65^+/+^ and CCDC65^+/-^ groups were treated with urethane (1 g/kg in 0.9% NaCl solution) intraperitoneally for 20 weeks. **(b)** Lungs were collected 6 months after the last urethane treatment. **(c)** The number of tumor nodules. **(d)** The HE staining of the harvested lung samples. **(e)** The average area of tumor nodules.

Mice aged 6-8 weeks in the CCDC65^+/+^ and CCDC65^+/-^ groups were injected intraperitoneally once weekly with 1g/kg urethane for 20 weeks to induce a LUAD model (Fig. 2a). Twenty weeks after the end of the urethane injection, the mice lung tissues were harvested (Fig. 2b).

The results showed that the average number of tumor nodules in the urethane-induced LUAD CCDC65^+/-^ group (3.09±1.30) was greater than that in the CCDC65^+/+^ group (1.62±0.77) (Fig. 2c). Subsequently, these lungs were excised, paraffin-embedded and used for HE staining (Fig. 2d), as well as the average area of the LUAD/lung adenoma on the sections were calculated. The results showed that the average area of LUAD/lung adenoma on the sections from CCDC65^+/-^ mice (2.71±1.60 ×10^4^ μm^2^) was larger than that from CCDC65^+/+^ mice tissue sections (1.44±1.23 ×10^4^ μm^2^) (Fig. 2e).

### Overexpression of CCDC65 inhibited the cell cycle and attenuated proliferation of LUAD cells both *in vitro* and *in vivo*

The expression of CCDC65 in H1975 and A549 cells were lower compared with other LUAD cell lines. The two cell lines were selected for subsequent experiments. The lentivirus were used to engineer steady CCDC65 upregulated cells. Two siCCDC65 sequences were used to engineer CCDC65 knockdown cells. The efficiency of overexpression or knockdown was presented in supplementary materials (Supplementary Fig. S3a-c).

**Figure 3:**
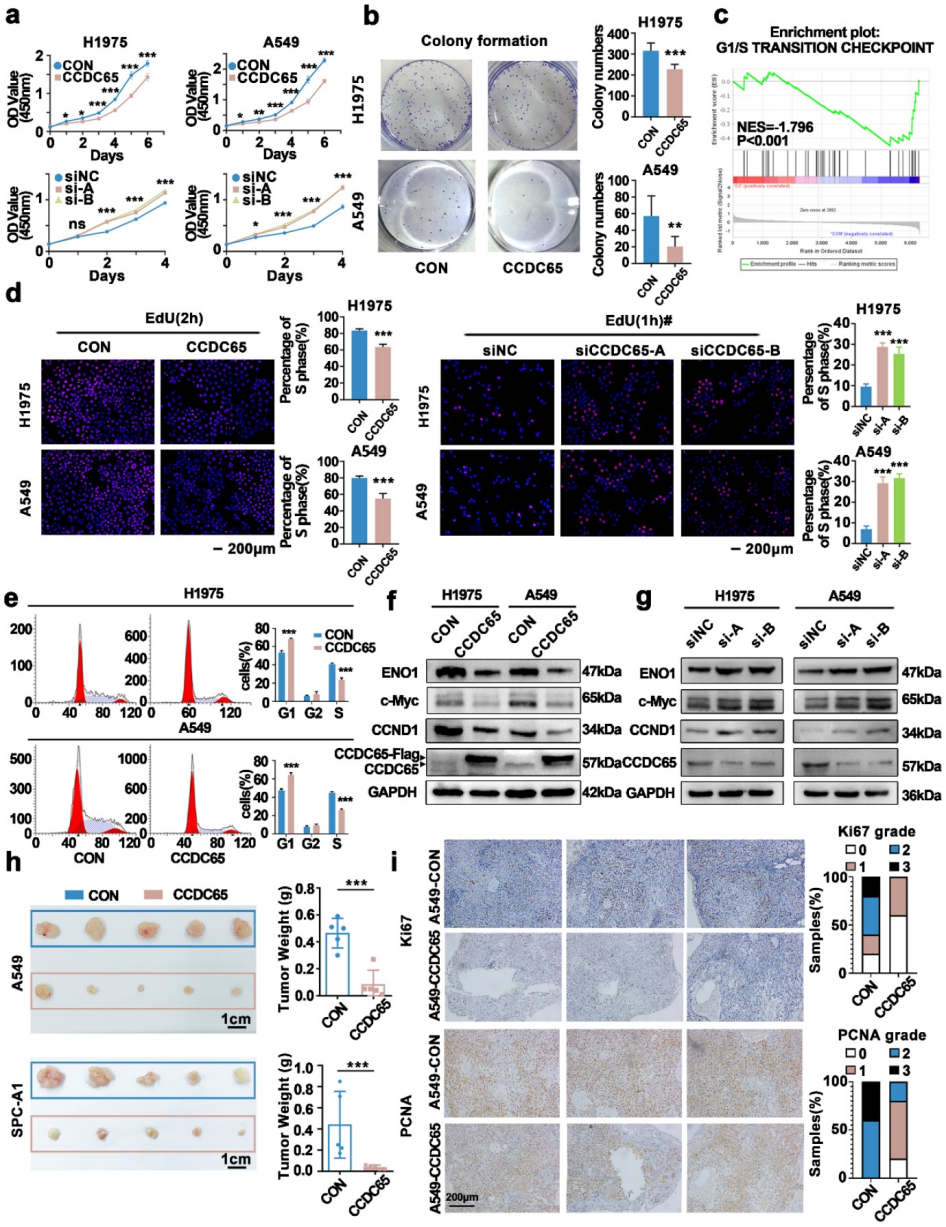
** CCDC65 inhibited H1975 and A549 cell proliferation and cell cycle transition, *in vitro* and *in vivo*. (a)** The establishment of CCDC65 over-expressing and knocking down cell lines. The effects of CCDC65 on the cell proliferation of H1975 and A549 cells were examined by **(a)** the CCK8 assay, **(b)** the Colony formation assay. **(c)** Enriched pathways based on the differential gene expression of cell lines (A549, SPC-A1 and 5-8F) whit CCDC65 or empty vector by using GSEA analysis. The effects of CCDC65 on the G1 to S transition of H1975 and A549 cells were examined by **(d)** EdU incorporation assay and **(e)** cell cycle flow analysis, Mean±SD (n=3). *P<0.05; **P<0.01.; ***P<0.005. #To prevent the phenomenon that the proportion of S phase cells were too high for us to do statistical analysis, we reduced incubation time in the knockdown experiment. **(f and g)** ENO1, c-Myc, CCND1 and CCDC65 were detected following transfection with CCDC65 lentivirus and siRNA. β-actin or GAPDH served as a loading control. **(h)** Excised tumors 28 days after implantation and measured the corresponding tumor volume. **(i)** Representative Ki67 and PCNA IHC staining of excised tumor tissues of A549-CON and A549-CCDC65 were shown.

The CCK8 assay was used to detect changes in cell proliferation in CCDC65 over-expressing cells. The results showed that, compared with the control group, CCDC65 over-expressing cells produced a lower absorption peak at 450 nm. Conversely, when compared with the control group, CCDC65-knockdown cells exhibited a higher absorption peak at 450nm (Fig. 3a). Colony formation showed that, compared with the control group, CCDC65 over-expressing cells produced less cell colony numbers (Fig. 3b).

Two lung adenocarcinoma cell lines A549, SPC-A1 and one nasopharyngeal carcinoma 5-8F were transfected with CCDC65 and empty vector and were analyzed by high-throughput sequencing. Gene Set Enrichment Analysis(GSEA) showed that CCDC65 was associated with multiple signaling pathways (Supplementary Table S3). The G1/S transition checkpoint pathway was screened out due to its relation to cell proliferation and most DEGs (differential expressed genes from high-throughput sequencing) were involved in it (Fig. 3c).

To further confirm the influence of CCDC65 on the G1/S transition, we evaluated the cell cycle by EdU assay. The results showed that the G1 to S cell cycle transition of cells were significantly inhibited in CCDC65 over-expressing cells, while the G1 to S cell cycle transition of cells were enhanced when CCDC65 was silenced (Fig. 3d). Next, Flow cytometry showed the higher G1 phase proportion and lower S phase proportion in CCDC65 over-expressing cells compared with control cells (Fig. 3e).

ENO1, which was reported to be regulated by CCDC65^18^, and the cell cycle-related proteins CCND1 and c-Myc were evaluated by western blotting. The data showed that ENO1, CCND1 and c-Myc were downregulated in CCDC65 over-expressing cells (Fig. 3f). To further confirm the effect of CCDC65, cell lines were transfected with siCCDC65, and the results showed that ENO1, CCND1 and c-Myc were upregulated in CCDC65 silenced cells (Fig. 3g).

Next, we performed an *in vivo* tumor formation experiment. Stably over-expressing CCDC65 cells and corresponding control cells were subcutaneously injected into the eight-week-old nude mice. Since the SPC-A1 cell line was used in the subsequent experiments, we selected SPC-A1 as one of the cell lines in the animal experiments. After implantation for 28 days, mice were sacrificed and the tumor nodules were harvested. The tumor nodules obtained were smaller in the CCDC65 group compared with that of the CON group (Fig. 3h). Ki67, PCNA immunohistochemical staining suggested that the proliferation of A549-CCDC65 cells were weaker than the A549-CON cells (Fig. 3i).

### CCDC65 interacted with c-Myc

In previous studies, we have confirmed that CCDC65 mediated the ubiquitination degradation of ENO1 by recruiting FBXW7. FBXW7, which acts as a critical tumor suppressor of human cancers, controls proteasome-mediated degradation of oncoproteins. However, c-Myc is one of those oncoproteins 26-28 and was inhibited by the overexpression of CCDC65 (Fig. 3f). Therefore, we attempted to explore the relationship between CCDC65, c-Myc and FBXW7.

**Figure 4:**
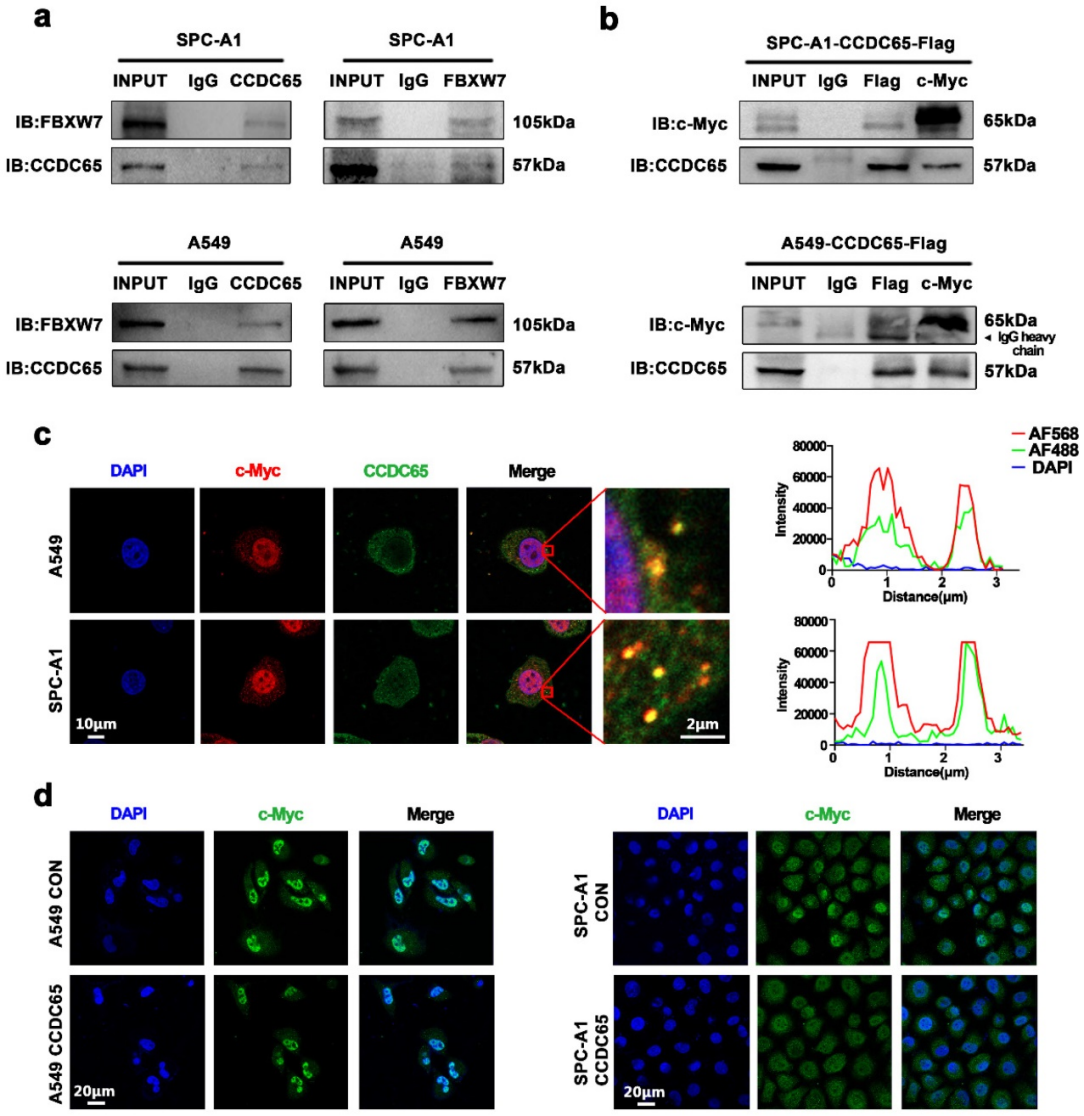
** CCDC65 interacted with c-Myc. (a)** Co-IP and western blotting assays indicated the interaction between CCDC65 and FBXW7. **(b)** Co-IP and western blotting assays indicated the interaction between CCDC65 and c-Myc. **(c)** The representative images of immunofluorescent staining of CCDC65 (green) and c-Myc (red). **(d)** Immunofluorescent staining showed CCDC65 reduced the expression and nuclear transportation of c-Myc.

Since the expression of CCDC65 in H1975 was significantly lower than that in other LUAD cells, SPC-A1 with relatively high expression of CCDC65 was selected for mechanism research. Firstly, Co-immunoprecipitation experiment was performed to verify the interaction between CCDC65 and FBXW7 in LUAD cells and the result confirmed their interaction relation. (Fig. 4a). And results also showed that CCDC65 interacted with c-Myc in both A549 and SPC-A1 cells (Fig. 4b). As a transcription factor, c-Myc was recognized as expressed in both cytoplasm and nucleus. Based on the IHC staining results shown before, CCDC65 was mainly expressed in the cytoplasm (Fig. 1i). Confocal microscopy experiments indicated that CCDC65 and c-Myc co-localized in the cytoplasm (Fig. 4c). In addition, immunohistochemical staining showed that the overexpression of the CCDC65 reduced the nuclear transportation of c-Myc in both A549 and SPC-A1 (Fig. 4d).

### CCDC65 mediated the ubiquitination degradation of c-Myc through the recruitment of FBXW7

**Figure 5:**
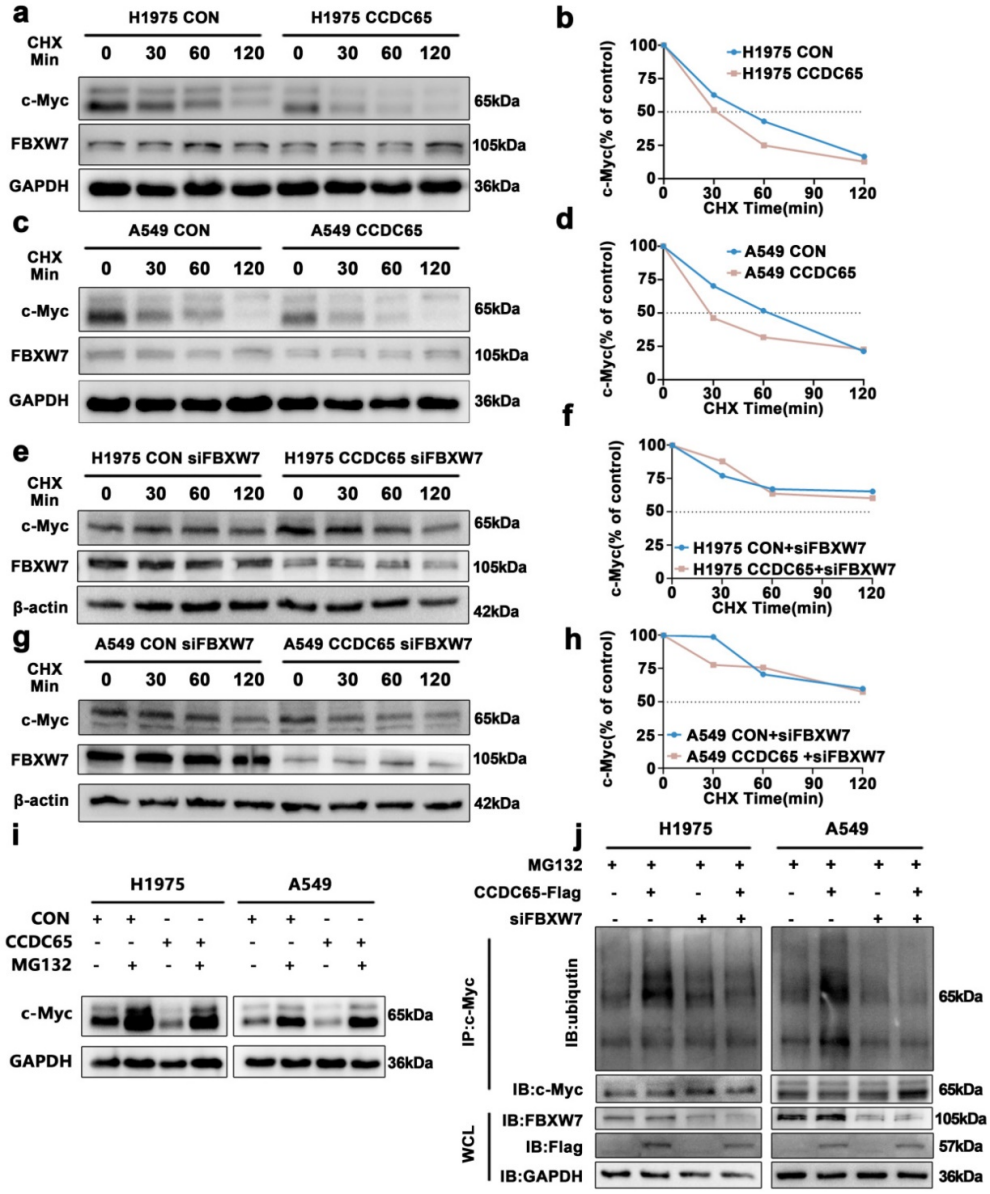
** CCDC65 mediated the ubiquitination degradation of c-Myc by recruiting FBXW7. (a, c)** CHX chase analysis of c-Myc protein half-life in CCDC65 over-expressing and control group in H1975 and A549 cells. CHX (50 µg/ml). **(e, g)** CHX chase analysis detected the effects of FBXW7 knockdown on protein stability of c-Myc. **(b,d,f,h)** The half-life curves of c-Myc protein in H1975 and A549 cells. **(i)** The effects of DMSO or MG132 (20 µM) treatment on the stability of the c-Myc protein in the control and CCDC65 overexpression groups. **(j)** Co-IP and western blotting assays were used to detect the effect of CCDC65 overexpression and treated with siFBXW7 on the ubiquitination level of c-Myc.

To further explore the influence of CCDC65 on c-Myc, ubiquitination research was performed. The CHX chase assay revealed that the c-Myc half-life in CCDC65 over-expressing cells had significantly shorter than that in control cells (Fig. 5a-d, Supplementary Fig. S3d, 3e). And the change of c-Myc half-life induced by CCDC65 was counteracted after being treated with siFBXW7 (Fig. 5e-h, Supplementary Fig. S3f, 3g). After treated with MG132 for 4 hours, the downregulation of c-Myc induced by CCDC65 was reversed (Fig. 5i, Supplementary Fig. S3h). Then the results of immunoprecipitation and western blotting determined that, depending on FBXW7, the overexpression of CCDC65 promoted c-Myc ubiquitination (Fig. 5j). These results suggested that CCDC65 mediated the ubiquitination degradation of c-Myc through the recruitment of FBXW7.

### c-Myc bound to the ENO1 transcriptional regulatory region and promoted its transcription

**Figure 6:**
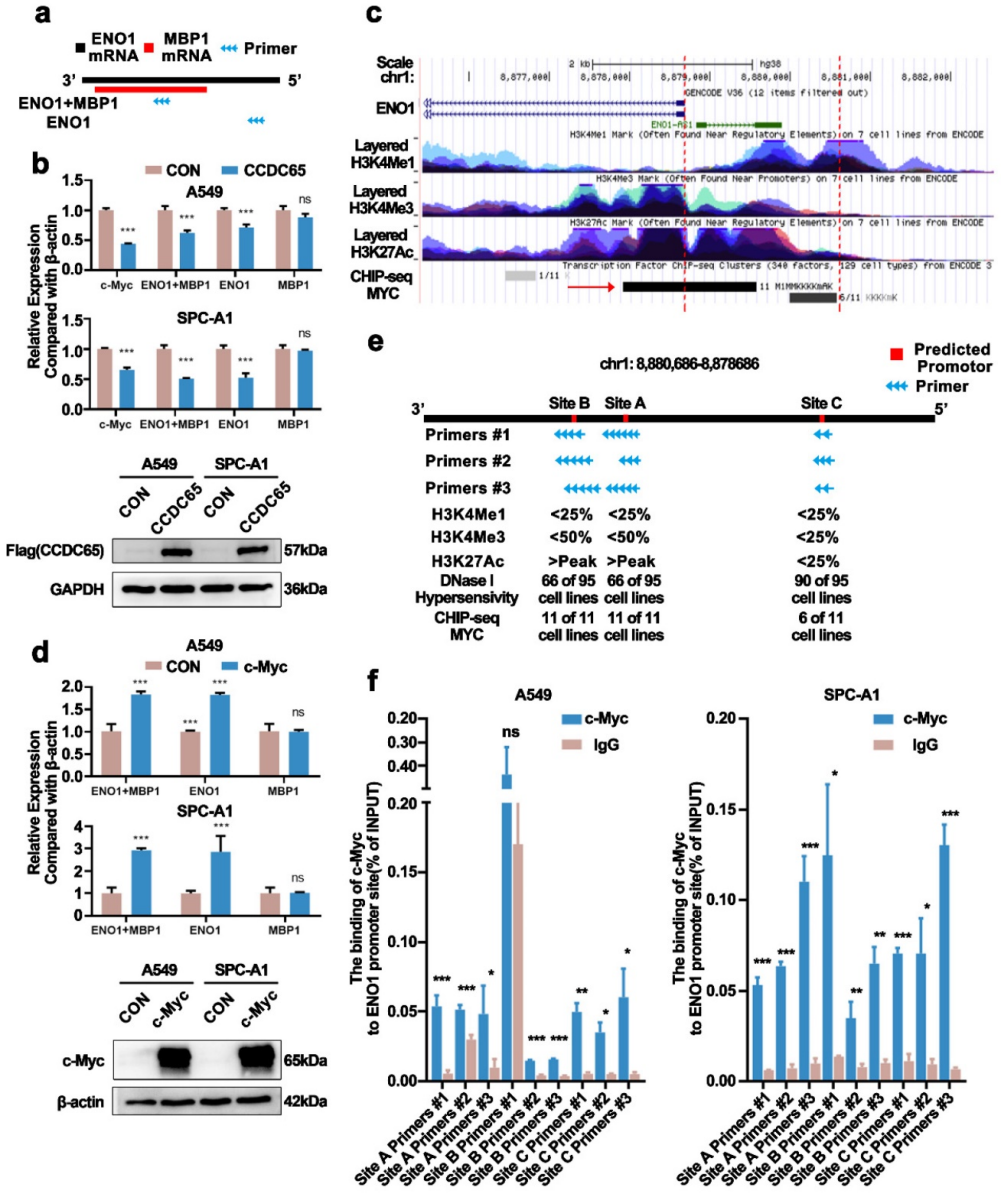
** c-Myc bound to the ENO1 transcriptional regulatory region and promoted its transcription. (a)** Diagram of the ENO1 specific primer sequence and ENO1/MBP1 common fragment primer sequence. **(b)** The overexpression of CCDC65 inhibited the transcription of c-Myc and ENO1. The expression of MBP-1 was calculated by (ENO1+MBP1)/ENO1. “ns” means no statistical significance. **(c)** The diagram of ENO1 in Genome Browser (http://genome.ucsc.edu/). The area between the red dotted lines means the upstream 2000bp of ENO1. **(d)** The overexpression of c-Myc promoted the transcription of ENO1. The expression of MBP-1 was calculated by (ENO1+MBP1)/ENO1. “ns” means no statistical significance. **(e)** The diagram of upstream 2000bp of ENO1. Red areas: Predicted promotor. Blue arrow: Primers designed according to the predicted promoter. **(f)** Ch-IP-qPCR detected the binding between c-Myc and the predicted promoter of ENO1.

In previous studies, we have confirmed that CCDC65 only downregulated the protein levels of ENO1 in gastric cancer 18. Myc promoter-binding protein-1 (MBP-1), a shorter protein variant of ENO1, was reported as a negative regulator of c-Myc expression 29. To eliminate the distractions MBP-1 probably brought about, we constructed ENO1 specific primer sequence (Fig. 6a). Nonetheless, we found that the mRNA levels of ENO1 and c-Myc were downregulated after the overexpression of CCDC65 in LUAD, which was different from CCDC65 in gastric cancer (Fig. 6b). Therefore, we speculated that CCDC65 inhibited ENO1 expression through different mechanisms. Strikingly, based on the transcription factor ChIP-Seq clusters (A549 cell line, ENCSR000DYC) from ENCODE (MYC) 30, 31 and UCSC Genome Browser, we found that there was a potential MYC binding region in the upstream of ENO1 coding region at 2000 bp (Fig. 6c). More details of MYC ChIP-seq clusters were presented in supplementary materials (Supplementary Table S4). Thus we transfected c-Myc into cells and found that the ENO1, instead of MBP-1, mRNA levels were upregulated (Fig. 6d). Combined with ChIP-seq and the result that c-Myc promoted the expression of ENO1 mRNA, we hypothesized c-Myc as a transcription factor binding to the ENO1 transcriptional regulatory region and regulating the expression of ENO1. Using the JASPAR database 32, we predicted the sequences that MYC might bind in 2000 bp upstream and 100 bp downstream of ENO1. The top 5 sequences were screened out (Supplementary Table S5). Nevertheless, among these 5 sequences, two sets of sequences were only a few bp apart, three sets of primers can be designed. In addition, we obtained the H3K4Me1, H3K4Me3, H3K27Ac marks on 7 cells (without lung adenocarcinoma cell lines) and DNase I Hypersensitivity clusters in 125 cell types (containing A549 cell line) from ENCODE (Supplementary Table S6) for predicting promotor region. The data showed that several cell lines had a high H3K27Ac mark on Site A and Site B and most of the cell lines were hypersensitive to being cut by the DNase enzyme on all sites (Fig. 6e). Chromatin immunoprecipitation assay and qPCR assay were performed and the results showed that both three sites were binding with c-Myc (Fig. 6f). ​Therefore, CCDC65 could inhibit ENO1 transcription by downregulating c-Myc. In addition, we tested whether CCDC65 mediated the ubiquitin degradation of ENO1. The results revealed that CCDC65 promoted the ubiquitin degradation of ENO1, which was in keeping with the previous studies (Supplementary Fig. S4).

### CCDC65 played the role of tumor suppressor by regulating the c-Myc/ENO1/AKT1 pathway

Several studies revealed that ENO1 promotes cancer phenotype by activating AKT signaling pathway 18, 33, 34. We observed the influence of CCDC65 on AKT1 phosphorylation and its downstream (Supplementary Fig. S5a). And the high-throughput mRNA sequencing between CCDC65^+/+^ and CCDC65^+/-^ mice normal lung tissues were conducted. Strikingly, KEGG pathway enrichment analysis suggested the AKT signaling pathway was affected by CCDC65 (Supplementary Fig. S5b, Supplementary Table S7).

We proposed that CCDC65 played the role of tumor suppressor by regulating the c-Myc/ENO1/AKT1 pathway. Rescue experiments were performed to test this hypothesis. C-Myc was introduced into stably over-expressing CCDC65 cells, CCK8 and EdU assays (Supplementary Fig. S6a,6b) showed that c-Myc reversed the cell cycle arrest and the change of cell cycle-related protein induced by CCDC65 (Supplementary Fig. S6c). When ENO1 was transfected into stably over-expressing CCDC65 cells, CCK8 and EdU assays(Supplementary Fig. S7a, S7b) were used to test changes in cell proliferation and cell cycle. The results showed that the effects on cell proliferation and cell cycle inhibited by CCDC65 were reversed after ENO1 overexpression. In addition, the western blotting showed that the expression levels of c-Myc and CCND1 inhibited by CCDC65 were also reversed (Supplementary Fig. S7c). Then, cells were treated with MK2206 (an AKT phosphorylation inhibitor) 35, 36 and siCCDC65. CCK8 (Supplementary Fig. S8a) and EdU assays (Supplementary Fig. S8b) results showed that the increase in cell proliferation induced by siCCDC65 was reversed after exposure to MK2206. CCND1, c-Myc, AKT1 and p-AKT1 were subsequently detected in treated cells, and the results showed that the up-regulation of CCND1 and c-Myc caused by siCCDC65 was also reversed (Supplementary Fig. S8c). To further clarify the relationship between CCDC65, ENO1, and c-Myc, the mRNA levels of which from the GDC TCGA LUAD database were analyzed. The results showed that, both in tumor tissues and normal tissues, CCDC65 was negatively correlated with ENO1 or c-Myc expression (Supplementary Fig.S9a). Meanwhile, ENO1 was positively correlated with c-Myc expression in tumors (Supplementary Fig.S9b). In addition, IHC was performed on the lung adenocarcinoma tissues from CCDC65 engineered mice (Supplementary Fig. S9c). After statistical analysis (Supplementary Fig. S9d) and protein correlation analysis based on the IHC score (Supplementary Fig. S9e-g), the results showed that ENO1 was strongly positively correlated with c-Myc, while ENO1 and c-Myc were negatively correlated with CCDC65. Because a large number of tissue slices had been used for HE staining in tumor area statistics, only a small amount of point-to-point tumor staining could be caught. Although the latter result was not statistically significant, the trends of those protein expression correlations could still be seen.

## Discussion

In the present study, we found that CCDC65, a member of the CCDC family, expressed in lower levels in LUAD tissues than in normal lung tissue based on the TCGA database. We also found the downregulation of CCDC65 mRNA expression in LUAD cell lines compared with normal bronchial epithelial cell lines. Consistent with the *in vitro* results from cell lines and the TCGA database, we found CCDC65 protein expression decreased in LUAD tissues compared with normal lung tissues. In addition, the expression of CCDC65 protein was negatively correlated with lymph node metastasis in LUAD patients. Furthermore, univariate Cox regression analysis demonstrated the downregulation of CCDC65 was an independent poor prognostic factor in LUAD patients. These data preliminarily suggested that CCDC65 exerted a tumor suppressive role in LUAD pathogenesis.

To clarify the role of CCDC65 in adenocarcinoma, we first engineered a CCDC65^-/-^ mouse model using CRISPR/Cas9 gene-editing technology. Serendipitously found that CCDC65^-/-^ mice had a larger head size, while their bodies were smaller and all CCDC65^-/-^ mice died within three weeks after birth. According to the sagittal plane of the whole brain and the known function of CCDC65 16, 17, we supposed the death of CCDC65^-/-^ mice was possibly relevant to hydrocephalus. But, this was not the point that we focused on, and in-depth research was not performed. Although CCDC65^-/-^ mice could not survive more than three weeks, there were neither phenotypic differences nor DFS differences between CCDC65^+/-^ mice and CCDC65^+/+^ mice. However, the mRNA and protein levels of CCDC65 in lung tissues of CCDC65^+/-^ mice were half as much as that in CCDC65^+/+^ mice. Hence, CCDC65^+/-^ and CCDC65^+/+^ mice were used as urethane-induced LUAD models. We found that urethane induced LUAD nodules were more numerous and larger in CCDC65^+/-^ mice than in CCDC65^+/+^ mice.

To further validate the tumor-suppressive activity of CCDC65, we established a CCDC65 over-expressing and knock-down human LUAD cell line model and used high-throughput sequencing for further research. The findings indicated that CCDC65 significantly inhibited the proliferation of LUAD cells *in vivo* and *in vitro.* GSEA showed G1/S transition checkpoint was the potential research direction. C-Myc and CCND1 were two key G1-S transition proteins involved in the regulation of the cell cycle 37-40. C-Myc was considered as one of the major driving factors of human tumorigenesis, which was essential for cell proliferation 41, 42. The mutation of c-Myc causes the death of mice and the knockdown of which significantly inhibits cell proliferation 43. As a transcription factor, c-Myc drives tumorigenesis through transcriptional regulation and CCND1 is one of the target genes regulated by c-Myc 44. ENO1 was reported to be inhibited by CCDC65 in gastric cancer and participated in the regulation of the cell cycle 18. Western blot showed the expression of c-Myc, CCND1 and ENO1 was downregulated by the overexpression of CCDC65. These data further supported that CCDC65 acted as a tumor suppressor via suppression of cell cycle G1-S transition signaling in LUAD.

In previous studies, we have confirmed that CCDC65 mediated the ubiquitination degradation of ENO1 by recruiting FBXW7 in gastric cancer 18. FBXW7, as a critical tumor suppressor of human cancers, controls proteasome-mediated degradation of oncoproteins. However, c-Myc is one of those oncoproteins 26-28 and was downregulated by the overexpression of CCDC65. Then we attempted to analyze the relationship between CCDC65 and c-Myc. The data showed that CCDC65 interacted with c-Myc and further mediated the ubiquitination degradation of c-Myc. And this degradation process depended on the FBXW7.

ENO1 is a multifunctional protein which implicated in critical biological progression pathways in cancers. As a glycolytic enzyme, ENO1 contributes to the Warburg effect and provides ATP to promote cancer development and progression 45. In addition, ENO1 promotes the plasminogen activation system and upregulates integrin to enhance the migration and invasion of cancers 46-48; ENO1 regulates gastric cancer cells stemness by stimulating glycolysis 49. Our previous studies found that ENO1 promoted tumor proliferation, migration, and invasion in non-small cell lung cancer 50. What conflicted with previous reports were that CCDC65 downregulated the mRNA levels of ENO1. ​Thus, we further explore how CCDC65 inhibited the transcription of ENO1. C-Myc was noticed because of its predicted binding to the upstream of ENO1 coding region by ChIP-seq and as a transcription factor which was downregulated by CCDC65. Moreover, ENO1 mRNA levels were upregulated after the introduction of c-Myc. Hence, we hypothesized that c-Myc was involved in ENO1 transcriptional regulation. Three potential ENO1 promoter binding sites were predicted by the JASPAR database and evaluated by the levels of H3K4me1, H3K4me3, H3K27ac, DNase I Hypersensitivity. Histone modification markers are often used as markers of transcriptional regulatory sites, such as H3K4me1 and H3K27Ac for enhancers or promoters, H3K4me3 for repressed regions 15, 51. DNase I hypersensitive sites are generic markers of regulatory DNA 52. Experimental results showed that c-Myc was separately bound to all three potential ENO1 promoter binding sites, which meant CCDC65 inhibited ENO1 mRNA levels by mediating c-Myc degradation. The current study reported a shorter protein variant of ENO1, which was named Myc promoter-binding protein-1 (MBP-1). MBP-1 is a negative regulator of c-Myc expression 53, 54. Our study proposed the transcriptional regulation of ENO1 by c-Myc, which complemented the precise regulation between ENO1 and c-Myc. Previous research reported that CCDC65 promoted ENO1 ubiquitination in gastric cancer 18. We also tested it in lung cancer and experiment results confirmed the interaction between CCDC65 and ENO1 and the overexpression of CCDC65 promoted the ubiquitination of ENO1.

CCDC65 downregulated ENO1 mRNA levels by decreasing the stability of c-Myc protein and downregulated ENO1 protein levels by mediating its ubiquitination degradation. Previous reports revealed that ENO1 could promote the phosphorylation of AKT1 33, 50. AKT1, as a member of the AKT family, plays a key role in multiple tumor signaling pathways, the phosphorylation of which promotes cell proliferation, cell cycle and cell steaminess 55-57. Our results also showed that CCDC65 decreased the phosphorylation of AKT1, but did not modulate total protein levels. Based on the different genes between CCDC65^+/+^ and CCDC65^+/-^ mice lung tissues, KEGG pathway enrichment analysis also supported that CCDC65 affected the AKT signaling pathway. The rescue experiments suggested that CCDC65 inhibited the proliferation of LUAD by inhibiting ENO1 and AKT1 phosphorylation.

In summary, this study showed that CCDC65 was downregulated in LUAD, which promoted the pathogenesis of LUAD based on evidence derived from *in vivo* and *in vitro* experiments. Molecular mechanism showed that CCDC65 recruited E3 ubiquitin ligase FBXW7 to induce the ubiquitination degradation of c-Myc, an oncogenic transcription factor in tumors, and reduced the binding between c-Myc and ENO1 promoter and thus suppressed the expression of ENO1 in mRNA levels. In addition, CCDC65 also recruited FBXW7 to degrade ENO1 protein through ubiquitinated modulation. The downregulated ENO1 further reduced the phosphorylation activation of AKT1, which thus inactivated the cell cycle signal. Our data demonstrated that CCDC65 was a potential tumor suppressor by recruiting FBWX7 to suppress c-Myc/ENO1-induced cell cycle signal in lung adenocarcinoma.

## Supplementary Material

Supplementary figures.

Supplementary tables.

## Figures and Tables

**Table 1:** Correlation between the clinicopathologic characteristics and expression of CCDC65 protein in lung adenocarcinoma

Characteristics	The expression of CCDC65		Chi square value	P value
	Low expression	High expression		
**Age**			0.000	0.994
≥50	47	27		
<50	7	4		
**Sex**			0.097	0.755
Male	33	20		
Female	21	11		
T stage*			0.155	0.694
1-2	38	20		
3-4	14	9		
N stage*			4.157	**0.041**
0-1	28	24		
2-3	16	4		
Clinical stage*			2.360	0.124
1-2	24	20		
3-4	21	8		

*Some patients are not included in the statistics due to the missing data.

**Table 2:** Summary of univariate and multivariate Cox regression analysis of overall survival duration

Characteristics	Univariate Cox			Multivariate Cox		
	Hazard ratio	95% CI	p value	Hazard ratio	95% CI	p value
Sex	0.92	0.534-1.584	0.763			
Age (y)	0.908	0.361-2.283	0.837			
Pathologic stage	0.987	0.586-1.664	0.961			
Lymph metastasis	3.22	1.517-6.835	**0.002**	1.78	0.439-73214	**0.419**
T stage	1.509	1.044-2.180	**0.028**	1.252	0.817-1.917	0.302
N stage	1.437	1.310-2.303	**<0.001**	1.336	0.822-2.170	0.242
Expression of CCDC65	0.551	0.306-0.993	**0.047**	0.513	0.264-0.998	**0.049**

## Abbreviations

CCD: Coiled-coil domain
CCDC65: Coiled-coil domain 65
ENO1: Alpha-enolase
PCD: Primary ciliary dyskinesia
SPF: specific pathogen-free
WT: Wild-type
HET: Heterozygous
KO: Knockout
CHX: Cycloheximide
NSCLC: Non-Small Cell Lung Cancer
LUAD: Lung adenocarcinoma
HR: Hazard ratio
CON: Control group
RT-qPCR: Reverse transcriptase-quantitative real-time PCR
IF: Immunofluorescence
Co-IP: Co-immunoprecipitation
Ch-IP: Chromatin-immunoprecipitation
