# Supplementary material for: CCDC65, a Gene Knockout that leads to Early Death of Mice, acts as a potentially Novel Tumor Suppressor in Lung Adenocarcinoma
Source: Int J Biol Sci. 2022 Jun 27;18(10):4171–86. doi: 10.7150/ijbs.69332 (PMC9274497; doi:10.7150/ijbs.69332)
Supplement: Supplementary file 1 — Supplementary figures. [file ijbsv18p4171s1.pdf]

Supplementary Fig. S1

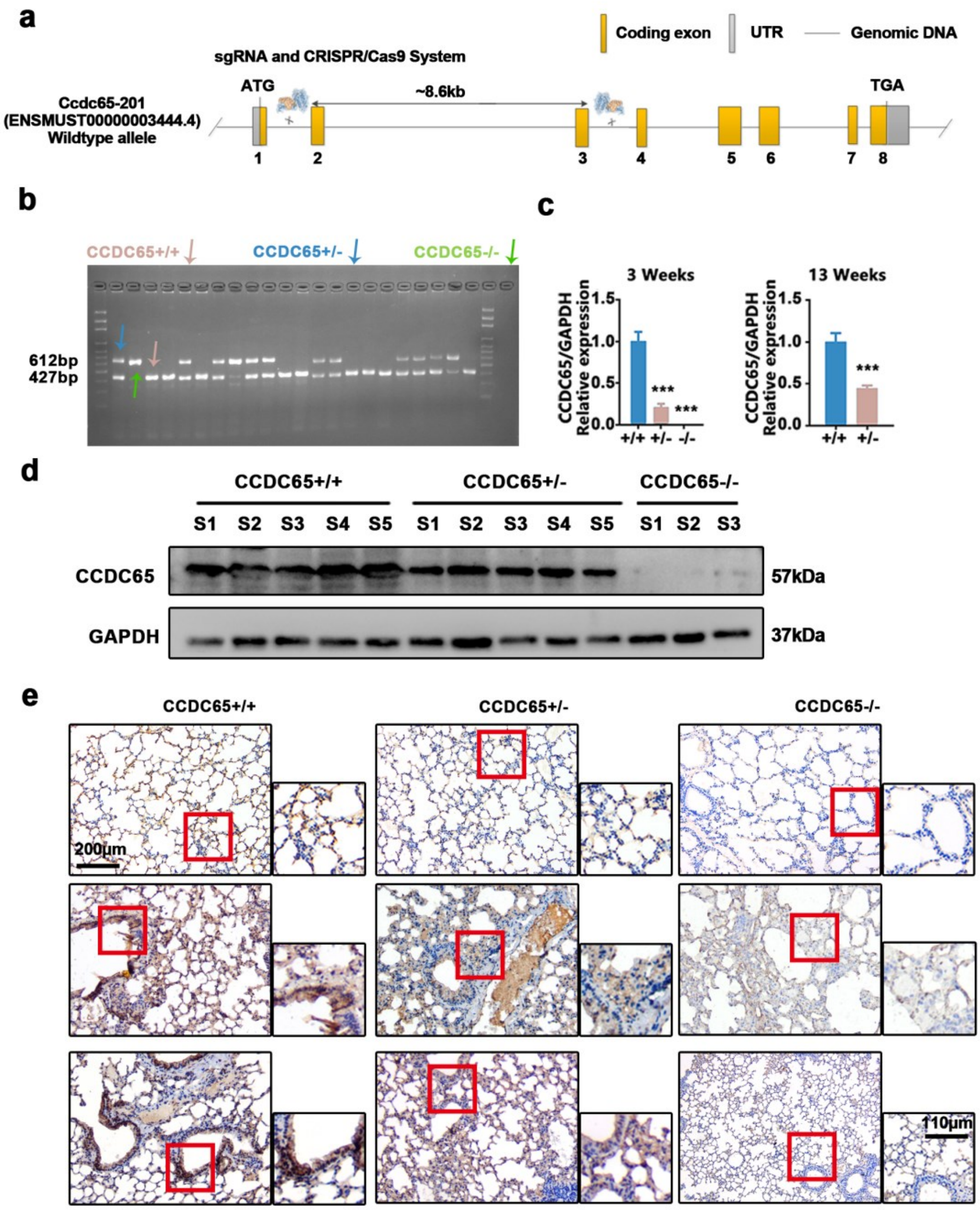

Figure S1 Identification of CCDC65 engineered mice. (a) The diagram of the CCDC65 engineered mice construction strategy. (b) DNA agarose gel electrophoresis was used to detect the genotype of the engineered mice. CCDC65$^{+/+}$ (Red arrow), CCDC65$^{+/-}$ (Blue arrow), and CCDC65$^{-/-}$ (Green arrow). (c) The mRNA expression of CCDC65 in different genotypes and different age engineered mice. (d) CCDC65 was detected by western blot. GAPDH served as a loading control. (e) Immunohistochemical staining was used in different genotype mice lung tissues.

Supplementary Fig. S2

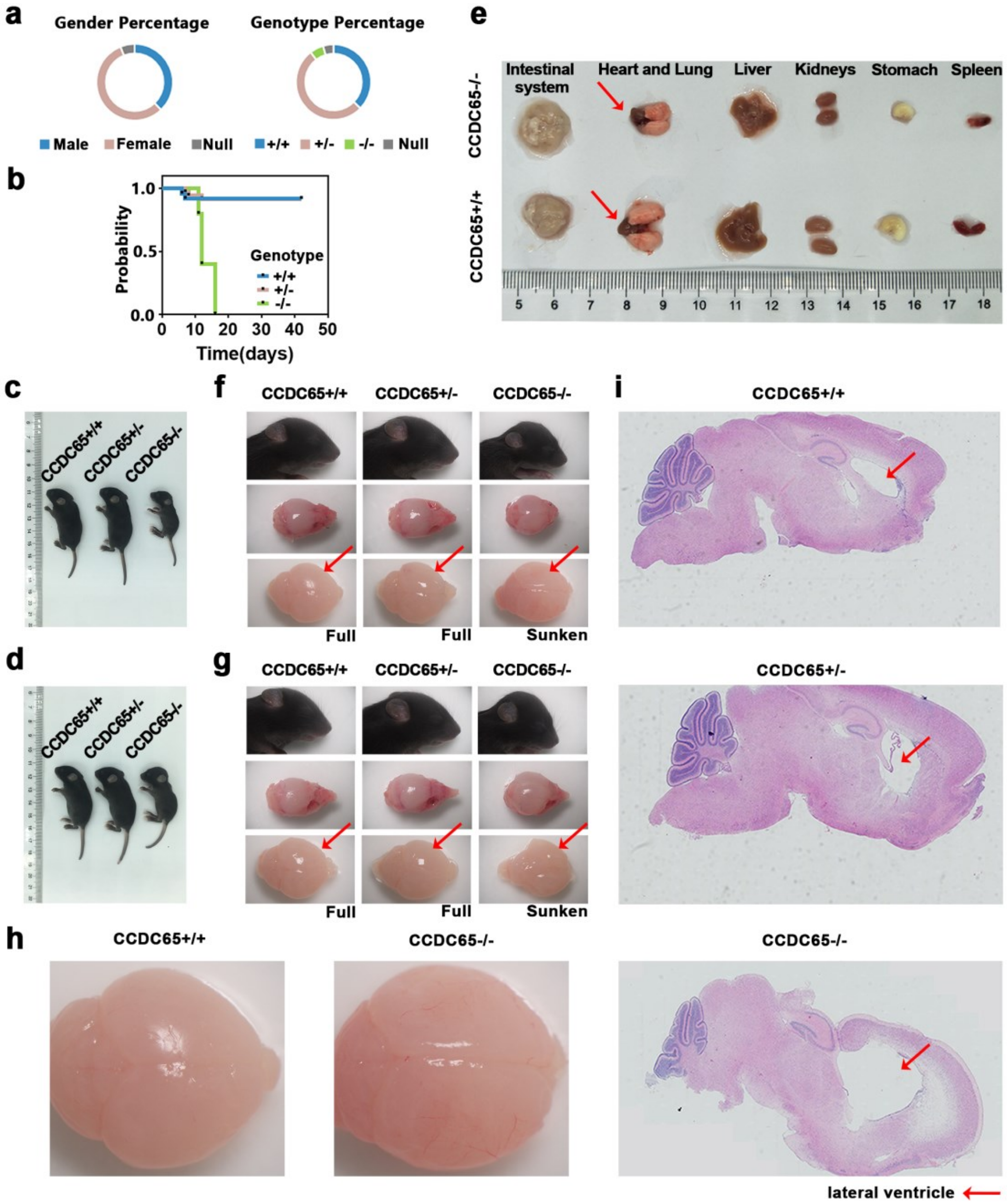

Figure S2 CCDC65$^{-/-}$ resulted in the disordered circulation of cerebrospinal fluid. (a) The percentage of sex and genotypes of mice (n=66). (b) The survival curves of CCDC65$^{+/+}$, CCDC65$^{+/-}$, and CCDC65$^{-/-}$ mice(n=66). (c) and (d) The body shape and head shape of CCDC65$^{+/+}$, CCDC65$^{+/-}$ and CCDC65$^{-/-}$ mice. (e) The visceral shape of the CCDC65$^{+/+}$ and CCDC65$^{-/-}$ mice. (f) and (g) The CCDC65$^{-/+}$ mice exhibited a larger head and smaller body compared with those of CCDC65$^{+/+}$ or CCDC65$^{+/-}$ mice. Models (f and g) were housed in different cages. (h) The image of enlarged brains of CCDC65$^{+/+}$ and CCDC65$^{-/-}$ mice. The brain of CCDC65$^{-/-}$ mice were sunken and the brain of CCDC65$^{+/+}$ or CCDC65$^{+/-}$ was enlarged. (i) Images

of HE staining of paraffin-embedded whole-mount sagittal sections of brains demonstrated significant expansion of lateral ventricle of CCDC65$^{-/-}$ mice. Red arrow: lateral ventricle

Supplementary Fig. S3

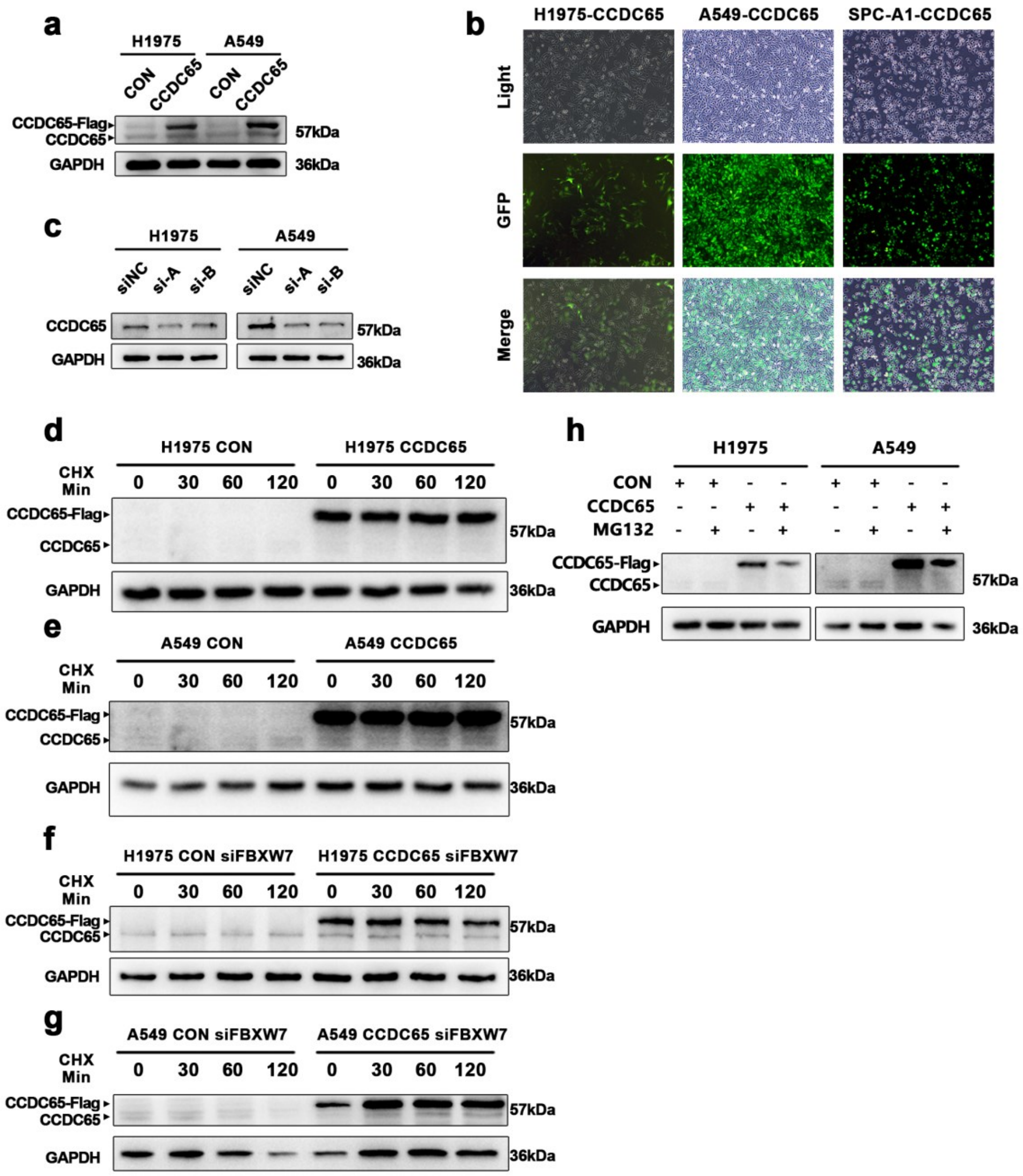

Figure S3 The efficiency identification of CCDC65 overexpression or knockdown. (a) CCDC65 overexpression efficiency was detected by western blot. The flag tag resulted in the increased molecular weight of CCDC65. The lower band was the original protein and the upper band was the overexpression CCDC65. (b) GFP confirmed the success of lentivirus infection with lung adenocarcinoma cells. (c) CCDC65 knockdown efficiency was detected by western blot. (d-h) CCDC65 overexpression efficiency (corresponding to Figure 5a-i).

Supplementary Fig. S4

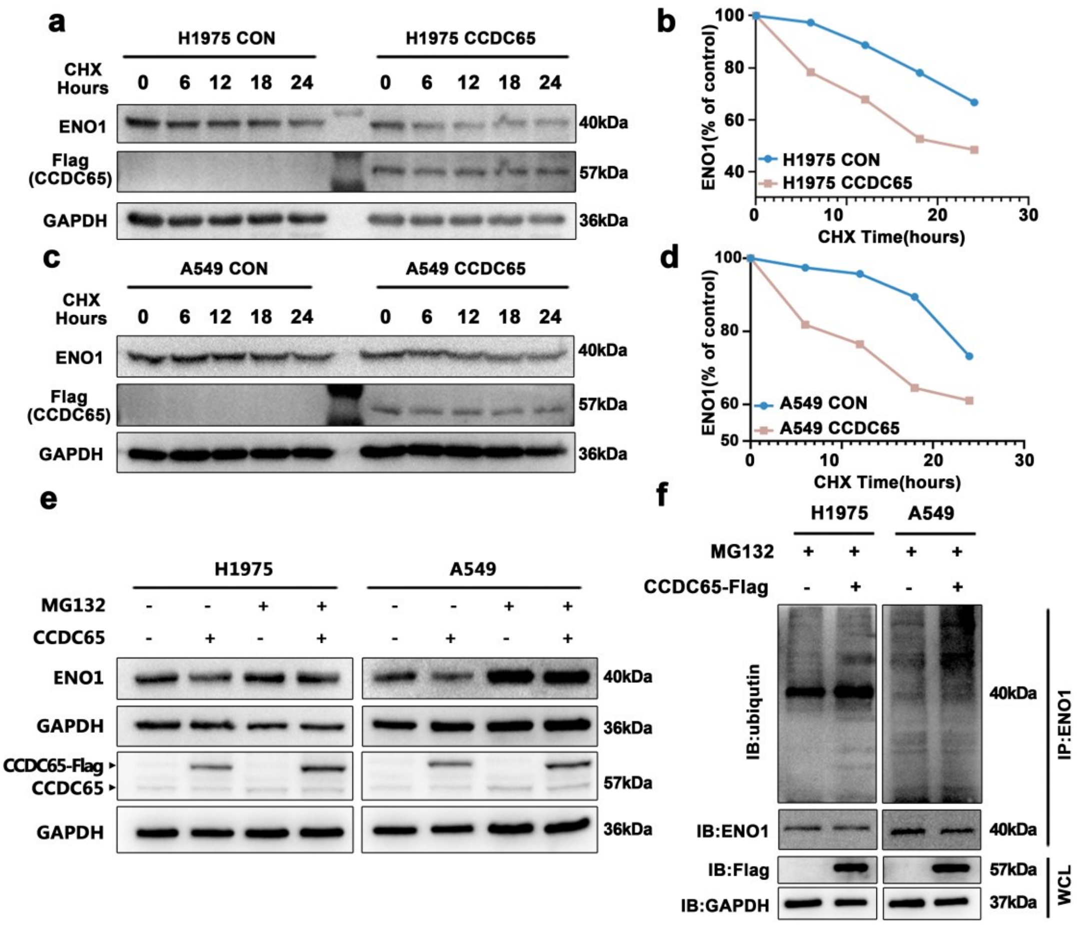

Figure S4 CCDC65 mediated the ubiquitination degradation of ENO1. (a)(c) CHX chase analysis of ENO1 protein half-life in CCDC65 over-expressing and control group in H1975 and A549 cells. CHX (50μg/ml). (b)(d) The half-life curve of ENO1 protein in H1975 and A549 cells. (e) The effects of DMSO or MG132 (20μM) treatment on the stability of ENO1 protein in the control and CCDC65 overexpression groups. (f)Western blotting detected the effects of CCDC65 overexpression on the ubiquitination level of ENO1.

Supplementary Fig. S5

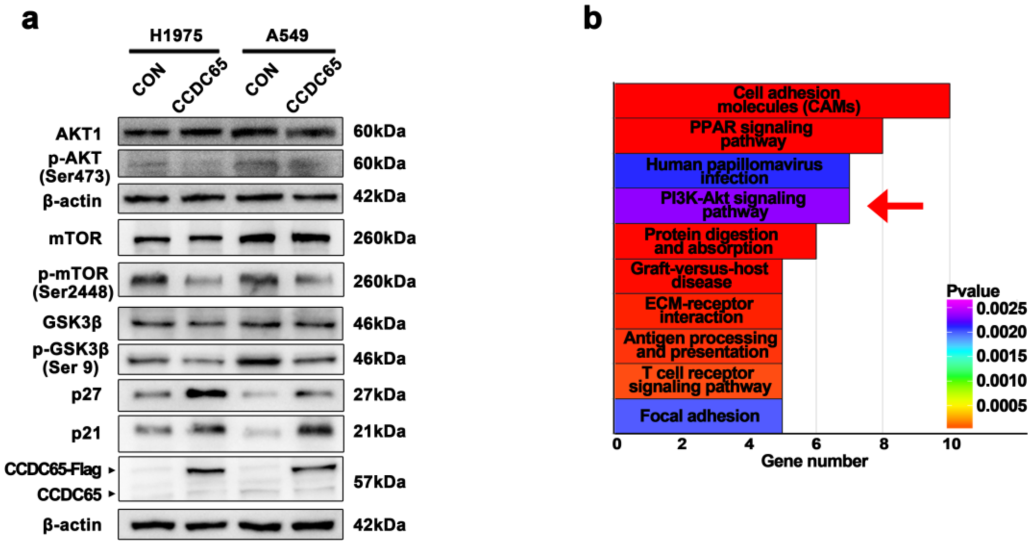

Figure S5 CCDC65 inhibited AKT1 phosphorylation. (a) The overexpression of CCDC65 inhibited the activation of AKT as well as its downstream such as p-mTOR(ser2448) and p-GSK3β(ser9), as well as promoted the expression of tumor suppressor genes such as p21and p27. β-actin served as a loading control. (b) KEGG signaling pathways enrichment was based on the different mRNA expression genes between CCDC65+/+ and CCDC65+/- mice normal lung tissues.

Supplementary Fig. S6

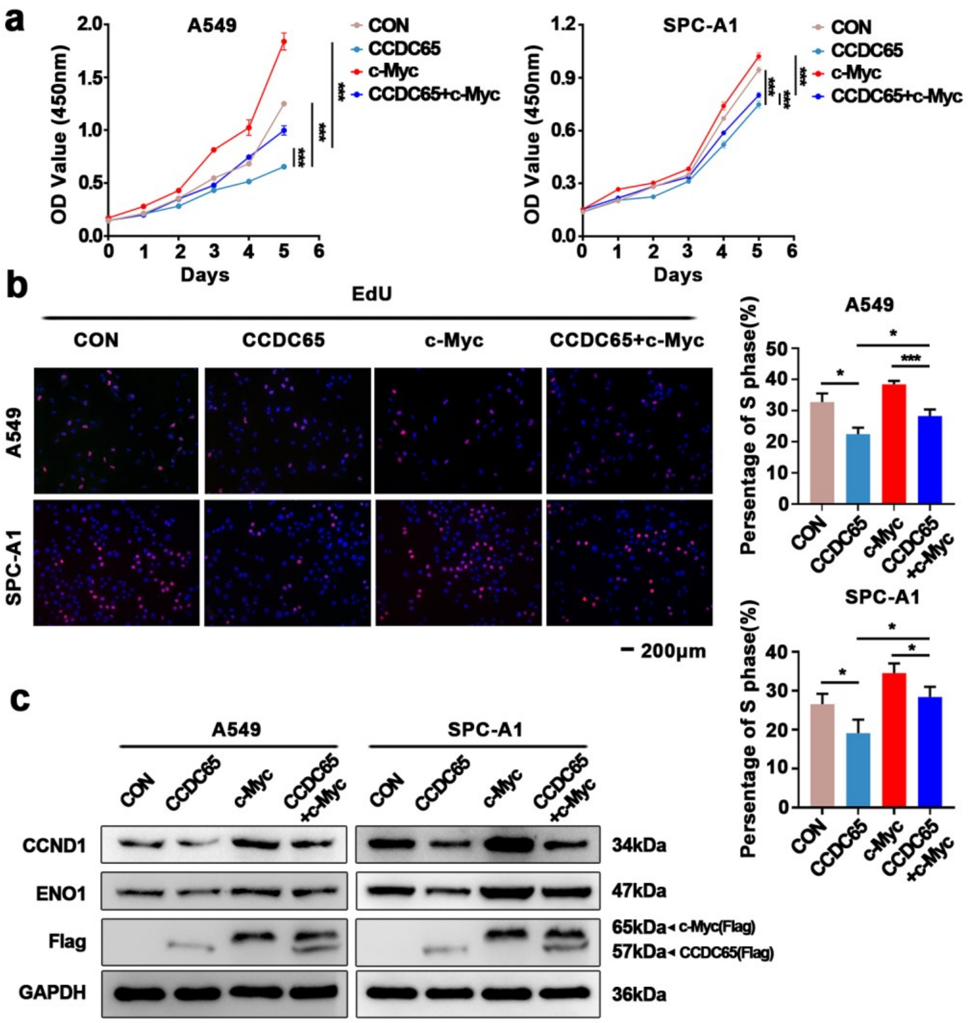

Figure S6 The overexpression of c-Myc reversed the inhibition of cell proliferation and cell cycle induced by CCDC65 introduction. The effects of c-Myc on the growth of H1975 and A549 cells stably overexpressing CCDC65 were examined by the (f)CCK8, (g)EdU incorporation assays. Mean±SD (n=3). (h) Western blotting was used to detect the change of CCND1, c-Myc and ENO1 after the introduction of c-Myc in CCDC65 stably overexpressing cells.

Supplementary Fig. S7

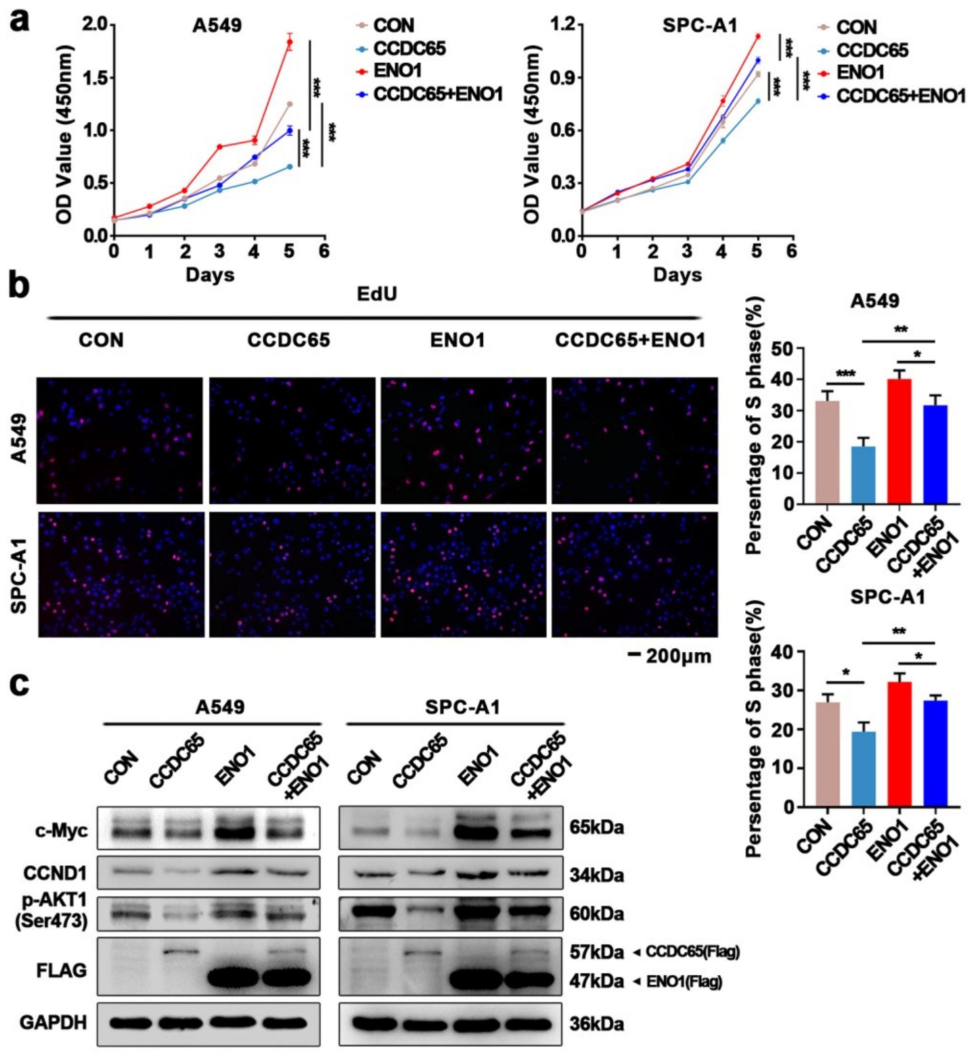

Figure S7 The overexpression of ENO1 reversed the inhibition of cell proliferation and cell cycle induced by CCDC65 introduction. The effects of ENO1 on the growth of H1975 and A549 cells stably overexpressing CCDC65 were examined by the (f)CCK8, (g)EdU incorporation assays. Mean±SD (n=3). (h) Western blotting was used to detect the change of c-Myc, CCND1, p-AKT1(ser473) and ENO1 after the introduction of ENO1 in CCDC65 stably overexpressing cells. “ns” means no statistical significance.

Supplementary Fig. S8

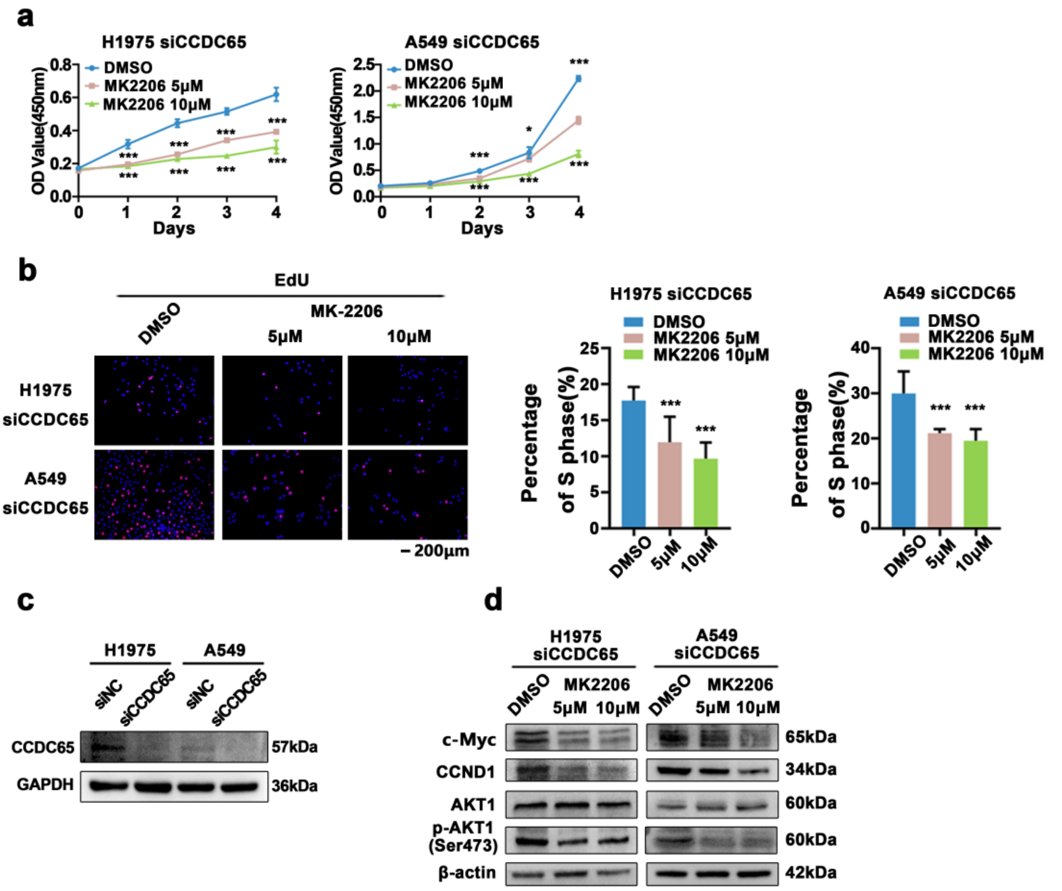

Figure S8 Inhibition of AKT1 phosphorylation reversed the cell proliferation and cell cycle induced by CCDC65 knockdown. MK-2206, an allosteric inhibitor of AKT, significantly inhibited the phosphorylation of AKT. CCDC65 knockdown cells were treated with DMSO, MK-2206 (5 µM, 10 µM), and then cell function was assessed by the (a) CCK8, (b, c) EdU incorporation assays. (e) Western blotting detected protein levels of c-Myc, CCND1, AKT1 and p-AKT1(ser473) in cells treated with DMSO, and MK-2206 (5 µM, 10 µM) respectively.

Supplementary Fig. S9

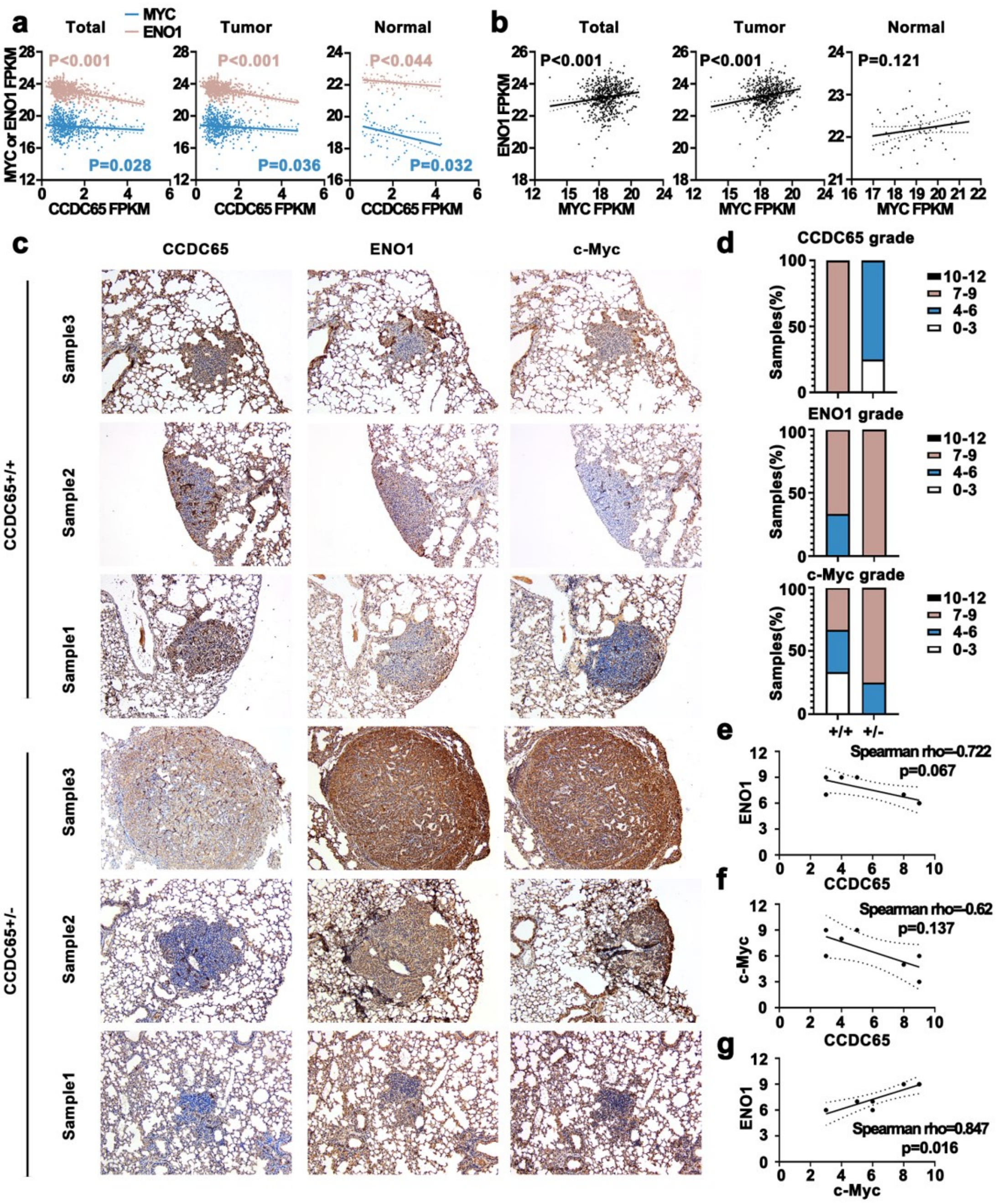

Figure S9 The expression correlation of CCDC65, ENO1 and c-Myc in TCGA database and CCDC65 engineered mice induced lung adenocarcinoma/adenoma tissues. (a) mRNA expression correlation between CCDC65 and ENO1/c-Myc.(b) mRNA expression correlation between ENO1 and c-Myc. The data of mRNA expression of (a) and (b) were from the GDC TCGA LUAD database. (c) The expression of CCDC65, ENO1 and c-Myc was tested by immunohistochemical staining. (d) The grade of CCDC65, ENO1 and c-Myc in CCDC65+/+ and CCDC65+/- mice was based on the IHC score. (e) Correlation between CCDC65 and ENO1 expression. (f) Correlation between CCDC65 and c-Myc expression. (g)

Correlation between ENO1 and c-Myc expression.
